# Aux/IAA and ARF Gene Families in *Salix suchowensis*: Identification, Evolution, and Dynamic Transcriptome Profiling During the Plant Growth Process

**DOI:** 10.3389/fpls.2021.666310

**Published:** 2021-05-26

**Authors:** Suyun Wei, Yingnan Chen, Jing Hou, Yonghua Yang, Tongming Yin

**Affiliations:** ^1^Key Laboratory of Tree Genetics and Biotechnology of Educational Department of China, College of Forestry, Nanjing Forestry University, Nanjing, China; ^2^Key Laboratory of Tree Genetics and Sivilcultural Sciences of Jiangsu Province, College of Forestry, Nanjing Forestry University, Nanjing, China; ^3^College of Life Sciences, Nanjing University, Nanjing, China

**Keywords:** auxin signaling, Aux/IAA and ARF gene families, polyploidization events, dynamic transcriptome profiling, plant growth process

## Abstract

The phytohormone auxin plays a pivotal role in the regulation of plant growth and development, including vascular differentiation and tree growth. The auxin/indole-3-acetic acid (Aux/IAA) and auxin response transcription factor (ARF) genes are key components of plant auxin signaling. To gain more insight into the regulation and functional features of Aux/IAA and ARF genes during these processes, we identified 38 AUX/IAA and 34 ARF genes in the genome of *Salix suchowensis* and characterized their gene structures, conserved domains, and encoded amino acid compositions. Phylogenetic analysis of some typical land plants showed that the Aux/IAA and ARF genes of Salicaceae originated from a common ancestor and were significantly amplified by the ancestral eudicot hexaploidization event and the “salicoid” duplication that occurred before the divergence of poplar and willow. By analyzing dynamic transcriptome profiling data, some Aux/IAA and ARF genes were found to be involved in the regulation of plant growth, especially in the initial plant growth process. Additionally, we found that the expression of several miR160/miR167-ARFs was in agreement with canonical miRNA–ARF interactions, suggesting that miRNAs were possibly involved in the regulation of the auxin signaling pathway and the plant growth process. In summary, this study comprehensively analyzed the sequence features, origin, and expansion of Aux/IAA and ARF genes, and the results provide useful information for further studies on the functional involvement of auxin signaling genes in the plant growth process.

## Introduction

Hormones play a central role in regulating plant growth and development, in which auxin is arguably the most important signaling molecule (Wang et al., 2015; Weijers and Wagner, 2016). The core components of the auxin signaling pathway are transport inhibitor-resistant 1/auxin signaling F-box (TIR1/AFB) auxin receptors, auxin/indole-3-acetic acid (Aux/IAA) transcriptional repressors, and auxin response factor (ARF) transcription factors, which play important roles in auxin-mediated growth and development by controlling auxin-responsive transcription (Hagen and Guilfoyle, 2002; Liscum and Reed, 2002; Berleth et al., 2004). When cellular auxin concentrations are low, Aux/IAA proteins act as repressors to inhibit DNA-binding ARF transcription factors and thus regulate auxin response elements (AuxREs) (Ulmasov et al., 1997b; Tiwari et al., 2001, 2004; Guilfoyle and Hagen, 2007). When cellular auxin concentrations are high, auxin increases the affinity of TIR1/AFB for Aux/IAA, thereby triggering the ubiquitin-mediated degradation of Aux/IAA proteins via the SCFTIR1/AFB E3 ligase complex (Gray et al., 2001; Zenser et al., 2001); the degradation of Aux/IAA proteins in turn allows ARF-mediated, auxin-responsive gene transcription to occur (Paponov et al., 2008).

Auxin/indole-3-acetic acid proteins are considered to act as repressors of auxin-responsive gene expression by interacting with ARF transcription factors via shared C-terminal domains (Tiwari et al., 2004; Yamaguchi et al., 2013). Aux/IAA proteins consist of N-terminal ethylene-responsive element binding factor-associated repressor (EAR) motifs that interact with TOPLESS (TPL) to inactivate ARF function (domain I) (Hiratsu et al., 2003; Tiwari et al., 2003); a middle region (MR) that contains the conserved amino acid sequence GWPP (V/I), which acts as the contact site for TIR1/AFB to promote degradation (domain II); and C-terminal Phox and Bem 1 (PB1) dimerization domains that mediate both homodimerization and heterodimerization among Aux/IAA and ARF proteins (domains III and IV) (Kim et al., 1997; Guilfoyle, 2015). ARF proteins mediate the expression of auxin-responsive genes by binding to the TGTCTC-containing *cis*-regulatory AuxREs found in the promoters of primary/early auxin response genes (Ulmasov et al., 1997a, 1999b; Tiwari et al., 2003). ARFs contain an N-terminal B3 DNA-binding domain (DBD) flanked on either side by dimerization domains, followed by a variable MR that confers transcriptional activator or repressor activity and a conserved C-terminal dimerization domain (CTD) that contains a PB1 domain involved in oligomerization and Aux/IAA-ARF heterodimerization (Ulmasov et al., 1999b; Tiwari et al., 2003). ARF proteins from early land plants can be divided into three classes, among which class A ARFs with a characteristic glutamine (Q)-rich MR are classified as transcriptional activators, and the remaining ARFs are transcriptional repressors and can be further divided into class C miR160-targeted ARFs and class B ARFs (Ulmasov et al., 1999a; Tiwari et al., 2003; Finet et al., 2013).

Genetic and phylogenetic analyses indicated that the components of the auxin signaling pathway originated from charophytes, and Aux/IAA and ARF already existed in charophyte genomes (Wang et al., 2015). The liverwort *Marchantia polymorpha*, a representative of the earliest-diverging land plants, harbors the most minimal auxin response machinery, in which a single TIR1/AFB ortholog, a single Aux/IAA, and three different categories of ARFs (A–C) are encoded by the genome, suggesting that the functional diversification of ARFs occurred before that of Aux/IAA and TIR1/AFB in the early stages of plant evolution (Flores-Sandoval et al., 2015). The moss *Physcomitrella patens*, the lycophyte *Selaginella moellendorffii*, and angiosperms diverged from each other between 700 and 450 million years ago (Lang et al., 2008). The genomes of both *P. patens* and *S. moellendorffii* encode multiple Aux/IAA and ARF proteins involved in the primary auxin response and form elaborate networks of auxin signaling components (Rensing et al., 2008; Banks et al., 2011). Analyses of the auxin response systems of higher plants have shown that the relatively simple auxin signaling mechanism of non-seed plants has evolved into a central regulator of many essential and diverse developmental processes in flowering plants. Phylogenetic analysis of the Aux/IAA and ARF families has shown that the two families have expanded independently in most flowering plants, including *Arabidopsis* (Liscum and Reed, 2002), poplar (Kalluri et al., 2007), rice (Sato et al., 2001; Jain et al., 2006), etc., suggesting that the seed plants are capable of complex auxin responses. According to the ratios of non-synonymous to synonymous nucleotide substitutions (Ka/Ks) among auxin signaling genes, positive selection has been detected in flowering plants, which may be the driving force for the evolution of the auxin signaling system (Paponov et al., 2009).

Tree growth involves a series of dynamic and continuous processes and is achieved by cell expansion and division activity in the vascular cambium. The plant hormone auxin plays an important role in regulating secondary growth and wood formation (Bhalerao and Fischer, 2014). The measurement of auxin levels across vascular cambial tissues in woody plants revealed a radial auxin concentration gradient, which may regulate cambial activity and the differentiation of cambial derivatives by providing positional signals to cells in the plant tissues (Uggla et al., 1996, 1998; Tuominen et al., 1997). Auxin may exert its influence on wood formation via the components of its signaling pathway, as suggested by the changes in the expression of many auxin-responsive genes, including auxin signaling genes, in wood-forming tissues (Andersson-Gunnerås et al., 2006; Nilsson et al., 2008). For example, the cambial auxin gradient is correlated with an expression peak of auxin signaling Aux/IAA genes in the developing xylem of hybrid aspen (Moyle et al., 2002). Genetic evidence has shown that the *ARF5* (*MONOPTEROS*) gene triggers auxin-mediated signal transduction and vascular tissue formation, and loss-of-function *mp* mutants show a highly reduced leaf vein system and very little vascular tissue (Berleth and Jurgens, 1993; Przemeck et al., 1996). An *ARF7* loss-of-function mutant displays altered leaf expansion, lateral root formation, and hypocotyl phototropism (Harper et al., 2000; Wilmoth et al., 2005).

*Salix suchowensis*, a small shrub willow reaching sexual maturity at 1 year of age, is an important bioenergy tree species. The chromosome-scale genome of *S. suchowensis* has been released (Wei et al., 2020). Given the importance of genes mediating auxin signaling in plant growth and development processes, the objectives of this study were as follows: (1) comprehensively identify Aux/IAA and ARF genes in the *S. suchowensis* genome and elucidate their sequence characteristics, genomic distribution, gene structures, and protein composition; (2) analyze polyploidization events and phylogenetic relationships to gain insight into the origin, evolution, and divergence of Aux/IAA and ARF genes in land plants; and (3) profile the dynamic expression patterns of Aux/IAA and ARF gene in cambium tissues at different growth stages and determine the candidate Aux/IAA and ARF genes that might be involved in the regulation of plant growth. These results will provide useful information for further studies in *S. suchowensis* and other trees to elucidate the functional involvement of Aux/IAA and ARF genes in diverse growth and development processes.

## Materials and Methods

### Identification, Sequence Analysis, and Phylogenetic Construction of AUX/IAA and ARF Families

We identified candidate Aux/IAA and ARFs proteins in nine land plant species through three steps: first, we obtained the total proteins of *S. suchowensis* from *S. suchowensis* genome version 2.0 (Wei et al., 2020) and those of eight other species (*M. polymorpha*, *P. patens*, *S. moellendorffii*, *Amborella trichopoda*, *Oryza sativa*, *Vitis vinifera*, *Populus trichocarpa*, and *Arabidopsis thaliana*) from the Phytozome v13^1^; second, we used *A. thaliana* Aux/IAA and ARF proteins as queries in BLASTP (Camacho et al., 2009) searches with an *e*-value cutoff of ≤1e−10 for predicted proteins in the eight genomes; and third, we employed HMMER v3.2.1 software (Finn et al., 2011) to examine protein domains using the Hidden Markov Model (HMM) profile of Aux/IAA (PF02309), DBD (PF02362), and Auxin_resp (PF06507) fetched from the Pfam database^2^. Subsequently, the conserved domains of all obtained Aux/IAA and ARF protein sequences were further checked by using both the NCBI conserved domain database (CDD) (Marchler-Bauer et al., 2011) and Simple Modular Architecture Research Tool (SMART) (Letunic and Bork, 2018) to confirm each candidate protein as an Aux/IAA or ARF protein.

All confirmed amino acid sequences of AUX/IAAs or ARFs were aligned using the ClustalW program with default parameters (Larkin et al., 2007). Phylogenetic trees were constructed using MEGA X via the neighbor-joining (NJ) method with 1000 bootstrap iterations (Kumar et al., 2018) and were further visualized and edited using the Interactive Tree of Life (iTOL) v5.7 web tool (Letunic and Bork, 2019).

Information about the physical locations of all AUX/IAA and ARF genes in *S. suchowensis* was obtained from the genome annotation GFF file, and the results were visualized using Circos (Krzywinski et al., 2009).

### Analysis of AUX/IAA and ARF Genes Duplication and Evolution

To investigate duplicated genes in the whole genome, we identified all paralogous genes in nine genomes by running BLASTP searches with an *e*-value cutoff ≤1e−10 (Camacho et al., 2009). Segmental, tandem, proximal, and transposed duplications were analyzed using MCScanX-transposed software (Wang et al., 2013). We used KaKs_Calculator 2.0 to calculate Ka, Ks, and the Ka/Ks (ω) value between paralogous gene pairs with the YN model (Wang et al., 2010). Information on duplicated AUX/IAA and ARF genes was filtered from the result of the whole-genome duplication analysis.

### Plant Materials and Sample Collection

Long-term phenotypic observations of growth traits in the full-sib F1 family population showed that the *S. suchowensis* offspring clone “S3412” grew to the tallest height, while the clone “S328” grew to the shortest height during a full growth cycle. Specifically, clone “S3412” showed higher values of growth traits (stem height and ground diameter) than clone “S328” at each time point during growth. Therefore, to detect Aux/IAA and ARF genes involved in tree growth, these two contrasting progenies were selected to analyze the dynamic transcriptome profiles of SuIAAs and SuARFs during the willow growth process. In detail, clones “S3412” and “S328” were asexually propagated from 15 cm uniform woody cuttings in open air under a natural photoperiod and received daily watering. Based on the growth curve measured and fitted from annual progenies, we determined six sampling time points from the primary growth stage to the fast growth and stationary growth stages (Supplementary Figure 1). Specifically, mixtures of xylem, vascular cambium, and phloem were collected from the two clones, “S3412” and “S328,” at 45, 75, 135, 195, 240, and 270 days after planting. In total, 36 samples (two different genotypes, six time points, and three biological replicates) were immediately frozen in liquid nitrogen and stored at −80°C for total RNA extraction.

### RNA Isolation, Library Preparation, and Sequencing

For each sample, total RNA was isolated using TRIzol reagent (Invitrogen, Carlsbad, CA, United States). The RNA quality was monitored on a 1% agarose gel, and the concentration was determined using a Qubit RNA Assay Kit with a Qubit 2.0 Fluorometer (Life Technologies, Carlsbad, CA, United States). The messenger RNA (mRNA) and small RNA sequencing libraries were constructed with the TruSeq RNA Library Prep Kit and the TruSeq Small RNA Library Prep Kit for Illumina (Illumina, San Diego, CA, United States), respectively, following the manufacturer’s instructions. The quality of each library was determined using an Agilent 2100 Bioanalyzer (Agilent Technologies, Santa Clara, CA, United States), and they were sequenced on a high-throughput Illumina platform (Illumina, San Diego, CA, United States) with the inclusion of both mRNA sequencing (paired-end 150 bp) and small RNA sequencing (single-read 50 bp).

### Bioinformatic Analysis of Sequencing Data

The raw mRNA reads for each sample were filtered with Trimmomatic (Bolger et al., 2014) to discard low-quality reads, trim adapter sequences, and eliminate low-quality bases. The clean reads were then aligned to the *S. suchowensis* genome using STAR (Dobin et al., 2013). Raw counts for each gene were derived using featureCounts (Liao et al., 2014). The mRNA expression levels of genes were normalized to fragments per kilobase of transcript per million fragments mapped (FPKM) values. Differential gene expression analysis between the two mRNA libraries was performed using the R packages of DESeq2 (Love et al., 2014), and the genes with an adjusted *P* < 0.01 were considered differentially expressed. We applied weighted gene correlation network analysis (WGCNA) to analyze the co-expression patterns of the SuIAA and SuARF genes in all samples (Langfelder and Horvath, 2008). The module eigengene (ME) corresponding to the first principal component was calculated for each module.

After removing the contaminant reads (adapter, polyA, and low-quality sequences and reads shorter than 18 nt or longer than 30 nt), the remaining small RNA reads were compared against several different small RNA reference databases using Bowtie2 (Langmead and Salzberg, 2012) to filter repeat sequences, ribosomal RNA (rRNA), transfer RNA (tRNA), small nuclear RNA (snRNA), small nucleolar RNA (snoRNA), and other non-coding RNA (ncRNA). Conserved microRNAs (miRNAs) were identified by BLASTN (Camacho et al., 2009) searches against miRBase v22.1 (Kozomara et al., 2019). The miRNA expression levels in each sample were estimated according to the mapping results and normalized to the number of transcripts per million clean tags (TPM). Differentially expressed miRNAs between two samples were calculated using the DESeq2 R package (Love et al., 2014), and those miRNAs with an adjusted *P* < 0.01 were assigned as differentially expressed. Potential miRNA targets were identified using the psRNATarget web tool with the default parameters (Dai et al., 2018).

## Results

### Identification and Sequence Analysis of SuIAAs and SuARFs

**FIGURE 1:**
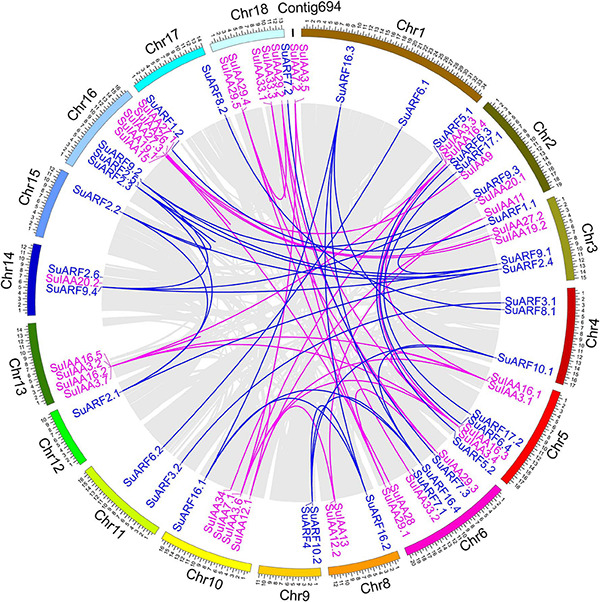
Genomic distribution of SuIAA and SuARF genes on the *Salix suchowensis* genome. The SuIAA genes and SuARF genes on each chromosome are plotted in purple and blue, respectively. Gray ribbons indicate collinear relationships among the blocks in whole genome; meanwhile, purple and blue links indicate the syntenic pairs of SuIAA genes and SuARF genes, respectively. The willow chromosomes and Contig694 are arranged with arcs with different colors, and the size of each arc is displayed in Mb.

**FIGURE 2:**
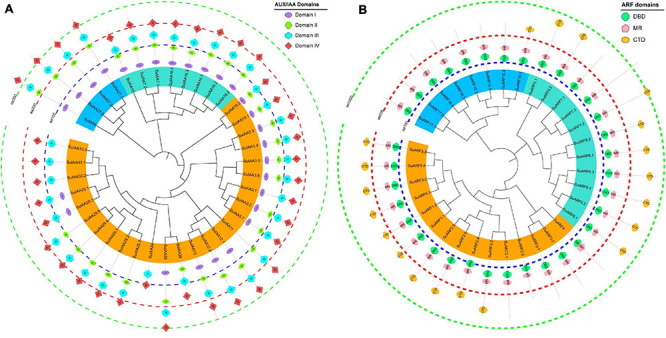
The phylogenetic relationships and conserved domain architecture of the SuIAA and SuARF proteins. **(A)** The phylogenetic tree of SuIAA proteins is divided into three subfamilies, which are represented by blue, cyan, and orange arcs. Domains I–IV are shaped with ellipse, vertical hexagon, horizontal hexagon, and rhombus from inside to outside. **(B)** The phylogenetic tree of SuARF proteins is also divided into three subfamilies, and canonical domains DBD (DNA-binding domain), MR (middle region), and CTD (C-terminal dimerization domain) are separately drawn with octagon, pentagram, and eclipse. Three dashed concentric circles of different colors are used to indicate the scale of the SuIAA and SuARF proteins length.

A total of 38 Aux/IAA genes were predicted in the *S. suchowensis* genome v2.0 (Figure 1 and Supplementary Table 2), which was equivalent to the 35 Aux/IAA genes previously predicted in the *P. trichocarpa* genome v1.1 (Kalluri et al., 2007) but slightly more than the 29 Aux/IAA genes found in the *A. thaliana* genome (Liscum and Reed, 2002). The length of the predicted SuIAA protein sequence is between 136 and 365 aa. All SuIAA genes were distributed on 10 chromosomes (Chr2, Chr3, Chr5, Chr6, Chr8, Chr10, Chr13, Chr14, Chr16, and Chr18) and one contig (Contig00694) in the *S. suchowensis* genome. The number of genes on each chromosome varied from 1 to 5, with the greatest number of SuIAA genes being found on chromosomes Chr2 and Chr16 and is the largest (Supplementary Table 1). The phylogenetic tree analysis of all SuIAA proteins showed that they form three subfamilies (Figure 2A). Most of the SuIAA proteins contained four conserved typical domains (I–IV). The SuIAA20.2 protein lacked domain II and may have a longer life cycle than other SuIAA proteins with domain II. Four SuIAA proteins (SuIAA34, SuIAA33.1, SuIAA33.2, and SuIAA33.3) lacked domains I and II, which may not play a role in transcriptional repression. Six SuIAA proteins (SuIAA28, SuIAA29.1, SuIAA29.2, SuIAA29.3, SuIAA29.4, and SuIAA29.5) also lack domain I. The SuIAA proteins lacking conserved domains were all distributed in one of the subfamilies (Figure 2A and Supplementary Table 1). All SuIAA proteins contained conserved domains III and IV, which might interact with ARFs to inhibit the expression of auxin-responsive genes. In addition, most SuIAA proteins contain two nuclear localization signals: a bipartite nuclear localization signal located between domains I and II and another typical nuclear localization signal located in domain IV (Supplementary Figure 2).

In total, 34 ARF genes were predicted in the *S. suchowensis* genome (Figure 1 and Supplementary Table 2), which was slightly fewer than the 39 ARF genes in the *P. trichocarpa* genome (Kalluri et al., 2007) and more than the 23 ARF genes in the *A. thaliana* genome (Liscum and Reed, 2002). The length of all SuARF protein sequences was between 581 and 1117 aa, and the average length of SuARF proteins was much greater than that of SuIAA proteins. With the exception of chromosomes Chr7, Chr13, and Chr19, SuARF genes were found on the remaining 16 chromosomes of the *S. suchowensis* genome. The number of SuARF genes on each chromosome ranged from 1 to 4, with Chr2 and Chr16 harboring the largest numbers of SuARF genes. Additionally, there were SuIAA gene clusters distributed on chromosomes Chr2 and Chr16, suggesting that the SuIAA and SuARF gene families might have evolved from common sites on ancient chromosomes. The phylogenetic analysis of the SuARF proteins in the *S. suchowensis* genome showed that they could be divided into three subfamilies (Supplementary Figure 3). The SuARF proteins in each subfamily contained the conserved DBD and MR domains, but 14 SuARF proteins lacked the CTD domain (Figure 2B and Supplementary Table 2), which might make them unable to interact with Aux/IAA and insensitive to auxin. Eleven SuARFs (SuARF5.1, SuARF5.2, SuARF7.1, SuARF7.2, SuARF7.3, SuARF8.1, SuARF8.2, SuARF6.1, SuARF6.2, SuARF6.3, and SuARF6.4) were found to harbor a glutamine (Q)-rich MR (Supplementary Figure 3), implying that these proteins are likely transcriptional activators.

### Phylogenetic Relationships and Polyploidization Events Reveal Aux/IAA and ARF Family Evolution

**FIGURE 3:**
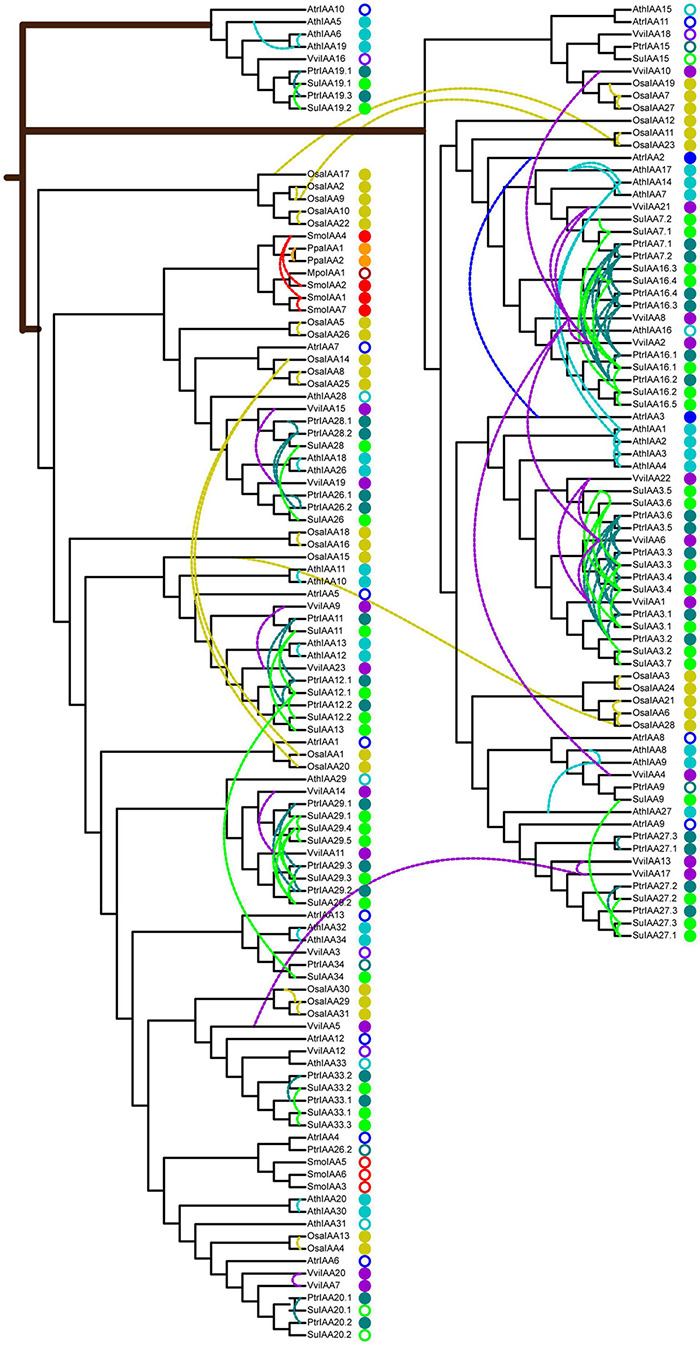
Phylogenetic relationships of auxin/indole-3-acetic acid (Aux/IAA) proteins in nine typical land plants. The neighbor-joining tree was constructed with one MpoIAA, two PpaIAA, seven SmoIAA, 13 AtrIAA, 31 OsaIAA, 23 VviIAA, 36 PtrIAA, 38 SuIAA, and 29 AthIAA proteins. The colored solid circles indicate duplicated genes in different species, and the colored rings indicate non-duplicated genes in different species. The colored dashed links represent paralog duplicated pairs. Mpo, *M. polymorpha*; Ppa, *P. patens*; Smo, *S. moellendorffii*; Atr, *A. trichopoda*; Osa, *O. sativa*; Vvi, *V. vinifera*; Ptr, *P. trichocarpa*; Su, *S. suchowensis*; Ath, *A. thaliana*.

To understand the evolutionary origin of auxin signaling in land plants, we constructed comparative phylogenetic trees of Aux/IAA and ARF sequences from eudicots (*A. thaliana*, *S. suchowensis*, *P. trichocarpa*, and *V. vinifera*), monocots (*O. sativa*), basal angiosperms (*A. trichopoda*), and lower eukaryotic plants (*S. moellendorffii*, *P. patens*, and *M. polymorpha*) (Table 1). The Aux/IAA phylogenetic tree was built with these 180 Aux/IAA protein sequences (Figure 3), which were classified into three main clades: clade A, clade B, and clade C. The Aux/IAA proteins in clade A can probably be traced back to the origin of early land plants because they include all Aux/IAA proteins present in the liverwort, moss, and lycophyte early plant lineages, suggesting that the Aux/IAA proteins in clade A functioned in early plant evolution. Clades B and C were found in basal angiosperms and in subsequent monocot and eudicot plant linages. In particular, clade C was a lineage-specific clade and contained a single-copy protein from basal angiosperms (*A. trichopoda*) and multiple-copy proteins from eudicots, which might be the product of Aux/IAA diversification in early angiosperms and thus constitute an alternative Aux/IAA lineage specific to eudicots.

**TABLE 1:** Summary of auxin/indole-3-acetic acid (Aux/IAA) and auxin response transcription factor (ARF) gene content from genomes of relevant taxonomic lineages.

**Species**	**Aux/IAA content (abbreviation)**	**ARF content (abbreviation)**	**Genome version**	**References**
*Marchantia polymorpha*	1 (MpoIAA)	3 (MpoARF)	v3.1	Flores-Sandoval et al., 2015
*Physcomitrella patens*	2 (PpaIAA)	15 (PpaARF)	v3.3	Rensing et al., 2008
*Selaginella moellendorffii*	7 (SmoIAA)	7 (SmoARF)	v1.0	Banks et al., 2011
*Amborella trichopoda*	13 (AtrIAA)	15 (AtrARF)	v1.0	This study
*Oryza sativa*	31 (OsaIAA)	25 (OsaARF)	v7.0	Sato et al., 2001; Jain et al., 2006
*Vitis vinifera*	23 (VviIAA)	21 (VviARF)	v2.1	Çakir et al., 2013; Wan et al., 2014
*Populus trichocarpa*	36 (PtrIAA)	37 (PtrARF)	v3.1	Kalluri et al., 2007
*Salix suchowensis*	38 (SuIAA)	34 (SuARF)	v2.0	This study
*Arabidopsis thaliana*	29 (AthIAA)	23 (AthARF)	v11	Liscum and Reed, 2002

All 180 ARF proteins from nine species (Table 1) could be classified into three main clades: clade A, clade B, and clade C (Supplementary Figure 4). The *M. polymorpha* genome encoded three ARFs (MpoARF1, MpoARF2, and MpoARF3) (Kato et al., 2015). Phylogenetic analysis showed that MpoARF1 belonged to clade A, which included activator ARF proteins such as AthARF5/MONOPTEROS, PtrARF5, and SuARF5. Clade B originated from an ancient branch, as this clade contained only five ARF proteins of lower eukaryotic plants, including MpoARF2 and four PpaARF proteins, which were gradually lost during evolution to higher plants. Phylogenetic analysis showed that MpoARF3 as well as the ARF10 and ARF16 proteins of *A. thaliana*, *S. suchowensis*, and *P. trichocarpa* were placed in clade C; these proteins may function as repressors and contain the target sequence of miRNA160. In summary, ARF proteins from land plants were phylogenetically classified into clades A–C, suggesting that two functionally divergent types of ARFs (MpoARF1 and MpoARF3) existed in the common ancestor of extant land plants.

Polyploidization events drive species evolution. We detected all segmental, tandem, proximal, and transposed duplicated genes in the genomes of nine species (Supplementary Figure 5). The proportions of duplicated genes in the nine species varied from 6.82 to 55.52%, among which the *M. polymorpha* liverwort genome contained the fewest duplicated genes, while more than 50% of the genes in Salicaceae genomes were involved in duplication events (Supplementary Figure 5 and Supplementary Table 3). These results were consistent with the ancestral polyploidization events experienced during land plant evolution. In the Aux/IAA and ARF phylogenetic trees, duplicated gene pairs were observed, indicating that these genes probably arose from gene duplication events (Figure 3 and Supplementary Figure 4). In the liverwort *M. polymorpha* genome, there was only one Aux/IAA gene and three ARF genes, which were not involved in gene duplication events. In the process of species divergence, the highest proportions of duplicated genes arose in the Aux/IAA and ARF gene families. For example, the proportions of duplicated Aux/IAA and ARF genes were 100 and 80% in monocots (*O. sativa*), while the average proportions of duplicated Aux/IAA and ARF genes were 86 and 70% in eudicots (*A. thaliana*, *S. suchowensis*, *P. trichocarpa*, and *V. vinifera*), respectively (Supplementary Figure 6). These results proved that the Aux/IAA and ARF genes shared ancestral polyploidization events and were amplified by whole-genome duplication during evolution.

To construct a potential evolutionary model of putative paralogous pairs of the Aux/IAA and ARF genes, we calculated the Ks values of all paralogous pairs in the whole genomes of nine species. Plotting the Ks values for segmental, tandem, proximal, and transposed paralog pairs clearly revealed that most land plants have experienced both ancestral polyploidization events and species-specific polyploidization events, except for *M. polymorpha*, whose characteristic of a low-duplicated gene content was similar to the situation in the genome of ancestral land plants (Supplementary Figure 7). Furthermore, the comparison of the Ks peak positions showed that Salicaceae species experienced the ancestral eudicot hexaploidization and the “salicoid” duplication before the divergence of poplar and willow occurred (Supplementary Figure 7). In the genomes of the nine species, most of the duplicated Aux/IAA genes originated via segmental duplication, which was particularly amplified in *A. thaliana*, *S. suchowensis*, and *P. trichocarpa*, while the proportions of transposed duplicated genes were increased in *V. vinifera*, *O. sativa*, and *S. moellendorffii* (Supplementary Table 4). The distribution of duplicated ARF genes was generally similar to that of the Aux/IAA duplicated genes, but the proportion of transposed duplicated genes was higher in *V. vinifera*, *O. sativa*, *A. trichopoda*, and *S. moellendorffii*. For example, almost all duplicated genes in *A. trichopoda* and *S. moellendorffii* were derived from transposed duplication, which might play an important role in altering gene functions and creating new genes (Supplementary Table 5). The Ks values further clarified the origination of these duplicated Aux/IAA and ARF genes through a specific polyploidization event. For example, the Ks values of the segmental duplicated Aux/IAA and ARF gene pairs in the *S. suchowensis* genome varied from 0.2 to 3.83 and 0.25 to 3.03, respectively, indicating that approximately half of the genes were derived from “salicoid” duplication, and the other half were generated by ancestral hexaploidization (Supplementary Tables 4, 5). The Ka/Ks values for all duplicated Aux/IAA and ARF pairs were <1 (Supplementary Tables 4, 5), suggesting that all duplicated Aux/IAA and ARF genes evolved mainly under the influence of purifying selection, with the loss of paralogous genes and limited functional divergence after the whole-genome duplications.

### Dynamic Expression Profiles of SuIAAs and SuARFs During the Growth Process

Tree growth is a result of cell expansion and division in the apical and cambial meristems, which are influenced by a variety of exogenous and endogenous factors, resulting in the formation of a complex regulatory network through the co-expression of plant hormones, functional genes, and transcription factors (Zhang et al., 2014). Numerous studies have shown the importance of the roles of Aux/IAA and ARF genes in the plant growth process (Przemeck et al., 1996; Moyle et al., 2002; Kalluri et al., 2007; Yu et al., 2015). To obtain insights into the roles of SuIAAs and SuARFs in the growth process, we examined the changes in the transcriptome profiles of SuIAAs and SuARFs using RNA-Seq technology. A mixture of cambium tissues was collected from the two contrasting clones, “S328” and “S3412,” at six growth stages (Supplementary Figure 8). We determined the time sequence of transcriptome expression to assess the expression patterns of the SuIAA and SuARF genes during plant growth (Supplementary Table 6 and Supplementary Figures 9, 10).

We carried out WGCNA to perform a co-expression analysis of the SuIAAs and SuARFs, resulting in the identification of six co-expressed gene modules, and each module eigengene was estimated to examine the expression pattern and assess its changes over time (Supplementary Figure 11). The expression patterns over a full growth cycle significantly showed that most SuIAAs and SuARFs presented the highest expression levels in the initial growth period (45 days after planting) or the stationary period (240 or 270 days after planting), while only a few SuIAAs and SuARFs showed the highest expression levels in the middle growth period (from 75 to 195 days after planting), suggesting that most Aux/IAA and ARF genes are involved in the activities of initial plant growth and dormancy.

**FIGURE 4:**
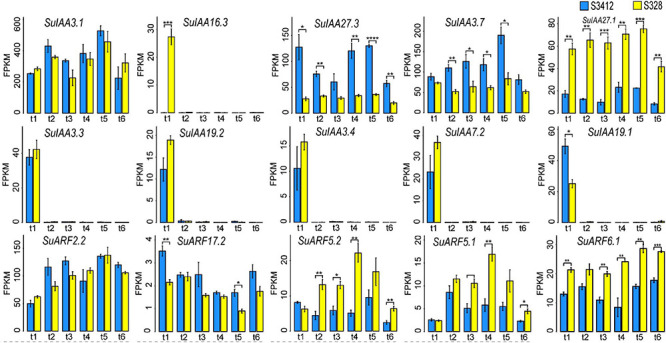
The time-sequential transcriptome profilings of some SuIAA and SuARF genes in two contrasting clones “S328” and “S3412.” The *X*-axis shows the different sampling time points (t1: 45 days after planting; t2: 75 days after planting; t3: 135 days after planting; t4: 195 days after planting; t5: 240 days after planting; and t6: 270 days after planting). The *Y*-axis represents the fragments per kilobase of transcript per million fragments mapped (FPKM) value, which are the mean ± SD of three replicates, and the *P*-values are shown as *P < 0.05, **P < 0.01, ***P < 0.001, and ****P < 0.0001.

The overall expression levels of SuIAA genes were much higher than those of SuARFs (Figure 4 and Supplementary Table 6). The *SuIAA3.1* and *SuARF2.2* genes were constitutively expressed throughout the growth process, and the FPKM values of *SuIAA3.1* and *SuARF2.2* were higher than 200 and 49, respectively, at different growth time stages. In the stationary period (240 days after planting), the expression of the *SuIAA3.1* and *SuARF2.2* genes reached the highest level, with FPKM values of 527.8 and 135.8, respectively. Furthermore, we found that the expression of five SuIAA genes (*SuIAA3.3*, *SuIAA19.2*, *SuIAA3.4*, *SuIAA7.2*, and *SuIAA19.1*) was induced in the initial growth period (45 days after planting), whereas they showed no expression in other growth stages, indicating that these genes were involved in plant primary growth. Generally, most SuIAA and SuARF genes showed similar time-sequential transcriptome profiles in “S328” and “S3412,” suggesting that they played similar roles in the cambiums of the two contrasting clones. However, some SuIAAs and SuARFs showed differential expression patterns in “S328” and “S3412,” including three genes (*SuIAA27.3*, *SuIAA3.7*, and *SuARF17.2*) that showed significantly higher expression levels in “S3412,” whereas four genes (*SuIAA27.1*, *SuARF5.2*, *SuARF5.1*, and *SuARF6.1*) showed lower expression levels in “S3412,” and these genes might play different roles in plant growth in the contrasting willow clones. Specifically, the *SuIAA16.3* gene was highly expressed only in “S328” in the initial growth period (45 days after planting). In summary, by analyzing the dynamic transcriptome profiles of SuIAA and SuARF genes in two contrasting clones, 15 candidate SuIAAs/SuARFs were found to participate in the regulation of willow growth (Figure 4).

Many studies have revealed that a number of ARF transcription factors are regulated by miRNAs (miR160 and miR167), among which ARF6 and ARF8 are targets of miR167, while ARF10, ARF16, and ARF17 are targets of miR160 (Mallory et al., 2005; Jones-Rhoades et al., 2006; Wu et al., 2006; Guilfoyle and Hagen, 2007; Liu et al., 2007). We identified six potential ssu-miR160s, two potential ssu-miR167s, and 14 SuARF targets of these miRNAs (Supplementary Figures 12, 13). According to the time-sequential expression profiling of SuARF targets and miRNAs, we found that ssu-miR160e–*SuARF10.2* and ssu-miR167e–*SuARF6.2* expression was in agreement with canonical miRNA–ARF interactions, suggesting that several miR160 and miR167 sequences may be involved in the regulation of plant growth by targeting ARF genes preferentially expressed in cambial cells (Supplementary Figures 12, 13).

## Discussion

The AUX/IAA and ARF genes are key components of the auxin signaling pathway, which plays important roles during plant growth and development (Ulmasov et al., 1997b; Hagen and Guilfoyle, 2002). In this study, a comprehensive set of 38 SuIAA and 34 SuARF proteins was identified (Figure 1), which was comparable to the numbers found in poplar (IAA, 36; ARF, 37) and were higher than the numbers found in *Arabidopsis* (29; 23), rice (31; 25), and grapevine (23; 21). Therefore, gene duplication may play an important role in a succession of genomic rearrangements and expansions (Vision et al., 2000). The features of the domains present in the AUX/IAA and ARF sequences provide useful information for the prediction of their functions (Tiwari et al., 2003, 2004). In this study, all SuIAA proteins contained conserved domains III and IV, which might form stable homodimers as well as heterodimers by interacting with ARFs to inhibit the expression of auxin-responsive genes (Figure 2A). A total of 10 SuIAA proteins did not contain domain I, suggesting that these genes lost the capacity to recruit TPL co-repressors and could not contribute to classical auxin signal transduction. In addition, five SuIAA proteins lacking domain II might not be rapidly degraded in the presence of basal or increased levels of auxin (Dreher et al., 2006). Through our RNA-seq analysis, we found that the expression levels of six SuIAA genes lacking domains I and II were extremely low throughout the growth process (Supplementary Table 6), suggesting that they exert little effect on plant growth and development. A typical ARF contains three domains, DBD, MR, and CTD (Ulmasov et al., 1999b; Tiwari et al., 2003). Here, all of the identified SuARF proteins had conserved domains DBD and MR, but 41.17% of the SuARFs were CTD-truncated ARFs (Figure 2B). The ARF members in *P. trichocarpa* (41.03%) included a similar percentage of CTD-truncated ARFs, while *Arabidopsis* shows a lower rate of CTD-truncated ARFs (21.74%) (Liscum and Reed, 2002; Kalluri et al., 2007), indicating that the CTD domain is relatively less conserved and that some auxin-responsive genes can be regulated in an auxin-independent manner (Ulmasov et al., 1997b). Based on the amino acid composition of MR domains, 11 SuARFs were identified as transcriptional activators, and the ratio of activators and repressors was 0.48, similar to that in poplar (0.54) and almost twice that in *Arabidopsis* (0.28), indicating a twofold enrichment of activator ARFs during Salicaceae evolution (Liscum and Reed, 2002; Kalluri et al., 2007).

Phylogenetic analysis of different Aux/IAA and ARF members from nine land plant species indicated that the Aux/IAA and ARF families underwent a number of polyploidization events and were amplified by whole-genome duplication during evolution (Figure 3 and Supplementary Figure 4). The presence of one Aux/IAA gene and three ARF genes in *M. polymorpha* indicated that Aux/IAA and ARF families dated back to close to the time of the origin of land plants (Flores-Sandoval et al., 2015). The Aux/IAA and ARF phylogenetic trees revealed high sequence similarity among land plants for the conserved evolution of these groups of genes resulting from a common origin and ancestors. Aux/IAA and ARF genes have been shown to be auxin regulated in early land plants, indicating that aspects of Aux/IAA and ARF functions have also been conserved in land plants (Imaizumi et al., 2002). Gene duplications result from genome rearrangement and expansion and play important roles in the diversification of gene functions (Cannon et al., 2004). The ancestors of duplicated Aux/IAA and ARF pairs might have originated before monocot–eudicot divergence (Wikström et al., 2001). Most of the duplicated pairs of Aux/IAA and ARF genes in Salicaceae appeared to have originated from the ancestral eudicot hexaploidization event and “salicoid” duplication that occurred before the divergence of poplar and willow (Supplementary Figure 7 and Supplementary Tables 4, 5). After the expansion of Aux/IAAs and ARF, the duplicated genes underwent an evolutionary process of purifying selection (Supplementary Tables 4, 5). Expression profiling of SuIAA and SuARF paralogs in growth stages showed functional redundancy and divergence during evolution (Supplementary Figures 9, 10), which is in accord with the observation that duplicated genes as well as sister pairs within a phylogenetic tree clade often display functional divergence (Kalluri et al., 2007; Paponov et al., 2009).

The expression profile of a gene family can provide clues about the functional diversification of different gene members. In this study, the time-sequential transcriptome profiles of the SuIAA and SuARF genes were investigated in cambium tissues at six growth times over a full growth cycle of willow plants. Dynamic expression profiling analysis showed that the number of SuIAAs and SuARFs with high expression levels in the initial growth stage was greater than that in the subsequent growth stages, suggesting crucial roles of the SuIAA and SuARF genes in early plant growth and development (Supplementary Figure 11). Specifically, *SuIAA3.3* and *SuIAA3.4* (orthologs of the *AthIAA3* gene), *SuIAA7.2* (ortholog of the *AthIAA14* gene), *SuIAA19.1*, and *SuIAA19.2* were inducibly expressed at 45 days after planting, with no expression in other growth stages (Figure 4). The *Arabidopsis* genes *AthIAA3* and *AthIAA14* have been reported to be components of the auxin signaling module (SLR/IAA14–ARF7–ARF19 and SHY2/IAA3–ARFs) regulating wood formation (Goh et al., 2012; Yu et al., 2015). Additionally, the poplar Aux/IAA gene *PtrIAA14.1* has been reported to regulate auxin signaling and vascular patterning in plant growth and development by interacting with ARF5 (Liu et al., 2015). Aux/IAA proteins regulate auxin-mediated gene expression via physical interactions with ARFs, so the preferential expression patterns of Aux/IAA genes and the complementary expression patterns to those of ARF genes may play a primary role in their physiological functions (Muto et al., 2007; Song et al., 2009). We found that the overall expression levels of SuIAAs were significantly higher than those of SuARFs (Supplementary Table 6) such as *SuIAA15*, *SuIAA16.1*, *SuIAA16.2*, *SuIAA16.5*, *SuIAA27.3*, *SuIAA3.1*, *SuIAA3.7*, and *SuIAA9*, and most of the orthologs of these genes in *Arabidopsis* and *Populus* also show high expression in xylem cells (Moyle et al., 2002; Kalluri et al., 2007; Nilsson et al., 2008), suggesting that these genes regulate cambium activity. We identified six co-expressed gene modules to assess the SuIAA and SuARF expression patterns and their changes over time in two contrasting clones (Supplementary Figure 11), and most of the SuIAAs showed expression patterns distinct from those of the SuARF genes (Supplementary Table 6), implying that SuIAA and SuARF genes are involved in specific plant growth processes with complex auxin signal transduction mechanisms. Some studies have reported that ARF5 and IAA12 in *Arabidopsis* may exhibit complementary regulatory functions in the control of embryogenesis and root meristem development in auxin signaling (Hamann et al., 2002). In addition, *PtrARF5* and *PtrIAA12* show contrasting expression patterns in roots and co-regulate root development in *Populus* (Kalluri et al., 2007). According to the time-sequential expression profiling of SuIAAs and SuARFs, we found that SuIAA12s (*SuIAA12.1* and *SuIAA12.2*) and SuARF5s (*SuARF5.1* and *SuARF5.2*) also presented complementary expression patterns: SuIAA12s was constitutively significantly highly expressed in “S3412,” while SuARF5s was constitutively significantly highly expressed in “S328” (Supplementary Table 6), indicating their putative involvement in mediating auxin responses during the plant growth process. Some SuARF genes, including *SuARF1.1*, *SuARF1.2*, *SuARF2.1*, *SuARF2.3*, *SuARF2.5*, *SuARF9.3*, and *SuARF9.4*, were significantly highly expressed at the stationary period (240 days after planting), which further confirmed that ARF1/2/9 could regulate leaf senescence, silique ripening, and floral organ abscission (Ellis et al., 2005). Transcriptome analysis of weeping and upright branches in another willow species (*Salix matsudana*) showed that two AUX/IAA genes and 10 ARF genes displayed differential expression and that those genes were highly likely to be responsible for the stem elongation and weeping traits of this species (Liu et al., 2017). By comparing the dynamic transcriptome profiles of orthologs of these genes in *S. suchowensis*, we found that the expression of *SuIAA3.4* was induced in the initial growth stage and that three orthologous SuARFs (*SuARF9.3*, *SuARF1.1*, and *SuARF5.2*) were differentially expressed in different willow growth stages, suggesting that some of these AUX/IAA and ARF gene orthologs may also be involved in regulating willow growth (Supplementary Figure 14).

The transcript abundance of ARFs might be regulated by miRNAs at the post-transcriptional level (Zhang et al., 2017). In *Arabidopsis*, miR167 controls the expression patterns of *AtARF6* and *AtARF8* to regulate flower development or lateral root formation and gravitropism (Nagpal et al., 2005; Gutierrez et al., 2009). Similarly, the regulation of *AtARF10* and *AtARF16* by miRNA160 has been implicated in seed germination, root cap formation, and *in vitro* shoot regeneration (Wang et al., 2005; Qiao et al., 2012; Liu et al., 2013). Our analyses revealed that the dynamic transcript levels of *SuARF10.2* might be regulated by miR160 (Supplementary Figure 12), while the *SuARF6.2* transcriptome profile is regulated by miR167 (Supplementary Figure 13), indicating that miRNA160/167 interact with ARF10/6 to carry out functions in plant growth and development.

## Conclusion

We identified 38 AUX/IAA and 34 ARF genes in *S. suchowensis* and established the classifications and evolutionary relationships of these genes through phylogenetic, gene structure, and conserved domain analyses. A phylogenetic analysis of nine land plants indicated that the Aux/IAA and ARF families in Salicaceae have undergone a number of polyploidization events and have been amplified via whole-genome duplication during evolution. Dynamic transcriptome profiling during the growth process revealed that some SuIAA and SuARF genes might participate in the regulation of plant growth, especially in the plant primary growth process. Additionally, miR160 and miR167 were predicted to post-transcriptionally regulate SuARF gene expression, suggesting that miRNAs are involved in the regulation of the auxin signaling pathway and the plant growth process. Taken together, our results provide a valuable resource for further studies on the biological functions of SuIAA and SuARF genes and the regulatory mechanisms of auxin-related pathways in the plant growth process.

## Data Availability Statement

The sequencing data presented in the study are deposited in the NCBI repository, accession number (PRJNA719970).

## Author Contributions

TY conceived and designed the research. SW analyzed the data and drafted the manuscript. YC and JH performed the experiments. TY and YY revised the manuscript. All authors reviewed and approved the final manuscript.

## Conflict of Interest

The authors declare that the research was conducted in the absence of any commercial or financial relationships that could be construed as a potential conflict of interest.

## Footnotes

1 https://phytozome-next.jgi.doe.gov/

2 http://pfam.xfam.org/

## Supplementary Material

Supplementary Figure 1 Growth trajectories of stem height for the full-sib F1 family population, n which the growth curve fitted using the logistic equation is shown in red.

Supplementary Figure 2 Amino acids sequence alignment and domain conservation analysis of SuIAA proteins. Four domains of SuIAA proteins were marked with different colors. Domain I contains an “LxLxL” motif. Between Domains I and II, a conserved “KR” motif was identified as a rate motif, and a bipartite nuclear localization signal (NLS) was located between the KR motif and Domain II. Domains III and IV together form type I/II Phox and Bem1p (PB1) domains. Another NLS was also observed in Domain IV.

Supplementary Figure 3 Alignment profile of conserved domains among the SuARF proteins. The shaded regions with different colors represent N-terminal B3-like DNA-binding domain (DBD), middle region (MR) domain, and C-terminal dimerization domain (CTD), respectively.

Supplementary Figure 4 Phylogenetic relationships of ARF proteins in nine typical land plants. The neighbor-joining tree was constructed with three MpoARF, 15 PpaARF, seven SmoARF, 15 AtrARF, 25 OsaARF, 21 VviARF, 37 PtrARF, 34 SuARF, and 23 AthARF proteins. The colored solid circles indicate duplicated genes in different species, and the colored rings indicate non-duplicated genes in different species. The colored dashed links represent paralog duplicated pairs. Mpo, *M. polymorpha*; Ppa, *P. patens*; Smo, *S. moellendorffii*; Atr, *A. trichopoda*; Osa, *O. sativa*; Vvi, *V. vinifera*; Ptr, *P. trichocarpa*; Su, *S. suchowensis*; Ath, *A. thaliana*.

Supplementary Figure 5 The proportion of duplicated genes in the genomes of nine species. Pro, proximal duplicated genes; Seg, segmental duplicated genes; Tan, tandem duplicated genes; Tra, transposed duplicated genes.

Supplementary Figure 6 Number of duplicated genes and non-duplicated genes of Aux/IAA and ARF in nine typical land plants. The colored bars indicate the number of duplicated genes in each species, and the colored rectangles indicate the number of non-duplicated genes in each species.

Supplementary Figure 7 The Ks values for segmental, tandem, proximal, and transposed duplicated genes in the genomes of nine species.

Supplementary Figure 8 Variations in the ground diameter and stem height of the two contrasting clones “S328” and “S3412” at six growth times. Values are the mean ± SD of three replicates. Letter ^∗^ represents significant difference at *P* < 0.05, while ^∗∗^ represents highly significant difference at *P* < 0.01 through *t*-test.

Supplementary Figure 9 Phylogenetic tree and time-sequential transcriptome profilings of duplicated SuIAAs. The colored dashed links in tree represent paralogous duplicated pairs of SuIAAs. Each histogram shows time-sequential transcriptome profilings of each duplicated SuIAA in two contrasting clones “S328” and “S3412.” The *X*-axis shows the different sampling time-points (t1: 45 days after planting; t2: 75 days after planting; t3: 135 days after planting; t4: 195 days after planting; t5: 240 days after planting; and t6: 270 days after planting). The *Y*-axis represents the FPKM values, which are the mean ± SD of three replicates, and the *P*-values are shown as ^∗^*P* < 0.05, ^∗∗^*P* < 0.01, ^∗∗∗^*P* < 0.001, and ^****^*P* < 0.0001.

Supplementary Figure 10 Phylogenetic tree and time-sequential transcriptome profilings of duplicated SuARFs. The colored dashed links in tree represent paralogous duplicated pairs of SuARFs. Each histogram shows time-sequential transcriptome profilings of each duplicated SuARF in two contrasting clones “S328” and “S3412.” The *X*-axis shows the different sampling time-points (t1: 45 days after planting; t2: 75 days after planting; t3: 135 days after planting; t4: 195 days after planting; t5: 240 days after planting; and t6: 270 days after planting). The *Y*-axis represents the FPKM values, which are the mean ± SD of three replicates, and the *P*-values are shown as ^∗^*P* < 0.05, ^∗∗^*P* < 0.01, and ^∗∗∗^*P* < 0.001.

Supplementary Figure 11 The expression patterns of the SuIAA and SuARF genes during plant growth. The change patterns of genes in each module were estimated with module eigengene (ME) values and displayed with histograms, in which six gray bars in each module represent normalized FPKM value, and the six digits represent different sampling time-points (t1: 45 days after planting; t2: 75 days after planting; t3: 135 days after planting; t4: 195 days after planting; t5: 240 days after planting; and t6: 270 days after planting). The heatmap shows FPKM expression of SuIAA and SuARF genes at different growth stages, in which different colors represent the range of transcriptome expression.

Supplementary Figure 12 The time-sequential expression profilings of ssu-miR160s and their ARF targets.

Supplementary Figure 13 The time-sequential expression profilings of ssu-miR167s and their ARF targets.

Supplementary Figure 14 The time-sequential expression profilings of differently expressed AUX/IAA and ARF genes in *Salix matsudana* and their homogeneous genes in *Salix suchowensis*.

Supplementary Table 1 Characteristics of Aux/IAA genes family in the *Salix suchowensis* genome v2.0.

Supplementary Table 2 Characteristics of ARF genes family in the *Salix suchowensis* genome v2.0.

Supplementary Table 3 The statistics of duplicated genes in the genomes of nine species.

Supplementary Table 4 The statistics of Ka, Ks, and Ka/Ks values for segmental, tandem, proximal, and transposed duplicated Aux/IAA genes in the genomes of *A. thaliana*, *P. trichocarpa*, *S. suchowensis*, *V. vinifera*, *O. sativa*, *A. trichopoda*, *S. moellendorffii*, and *P. patens*.

Supplementary Table 5 The statistics of Ka, Ks, and Ka/Ks values for segmental, tandem, proximal, and transposed duplicated ARF genes in the genomes of *A. thaliana*, *P. trichocarpa*, *S. suchowensis*, *V. vinifera*, *O. sativa*, *A. trichopoda*, *S. moellendorffii*, and *P. patens*.

Supplementary Table 6 The time-sequential transcriptome profiles of SuIAAs and SuARFs in the cambium tissues of two contrasting clones “S328” and “S3412.”
