# Supplementary material for: Aux/IAA and ARF Gene Families in Salix suchowensis: Identification, Evolution, and Dynamic Transcriptome Profiling During the Plant Growth Process
Source: Front Plant Sci. 2021 May 26;12:666310. doi: 10.3389/fpls.2021.666310 (PMC8188177; doi:10.3389/fpls.2021.666310)
Supplement: Supplementary Figure 1 — Growth trajectories of stem height for the full-sib F1 family population, n which the growth curve fitted using the logistic equation is shown in red. [file Data_Sheet_1.docx]

**Supplementary Figures**


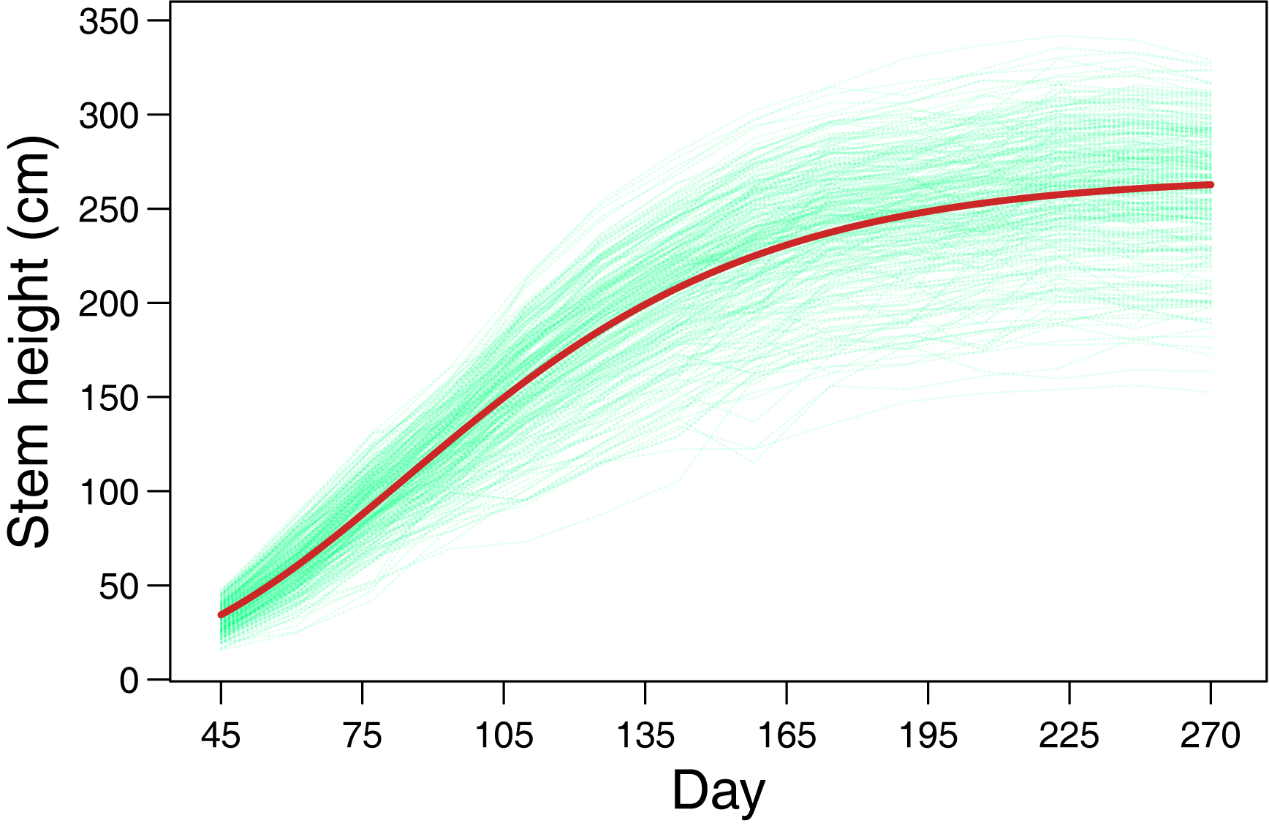


**Figure S1.** Growth trajectories of stem height for the full-sib F1 family population, in which the growth curve fitted using the logistic equation is shown in red.


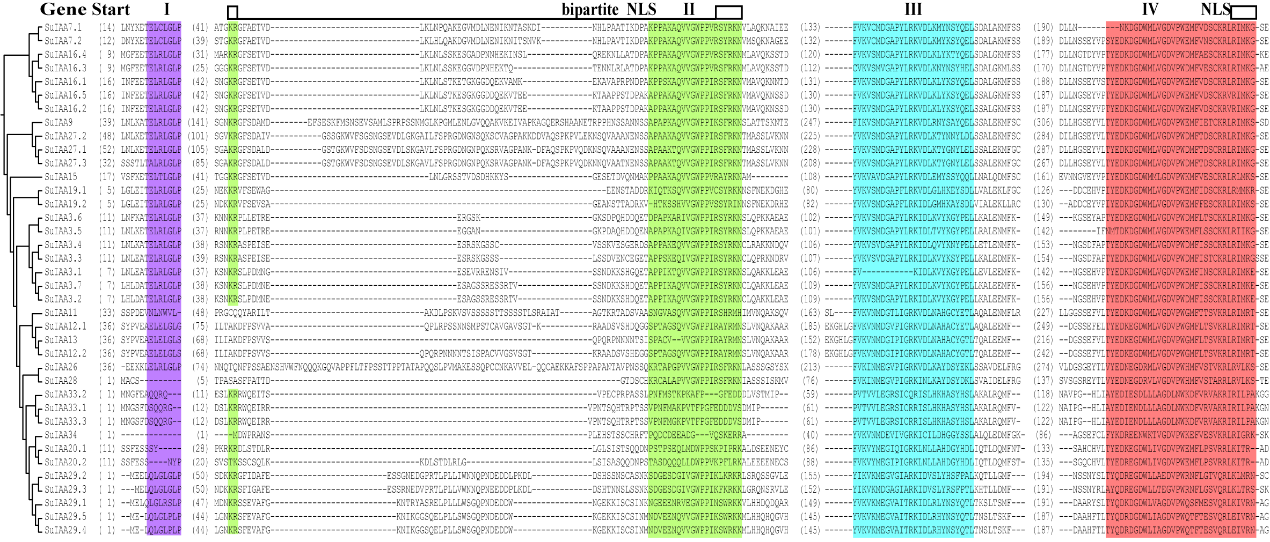


**Figure S2.** Amino acids sequence alignment and domain conservation analysis of SuIAA proteins. Four domains of SuIAA proteins were marked with different colours. Domain I contains an “LxLxL” motif. Between Domain I and II, a conserved “KR” motif was identified as a rate motif, and a bipartite nuclear localization signal (NLS) was located between the KR motif and Domain II. Domain III and IV together form type I/II Phox and Bem1p (PB1) domains. Another NLS was also observed in Domain IV.


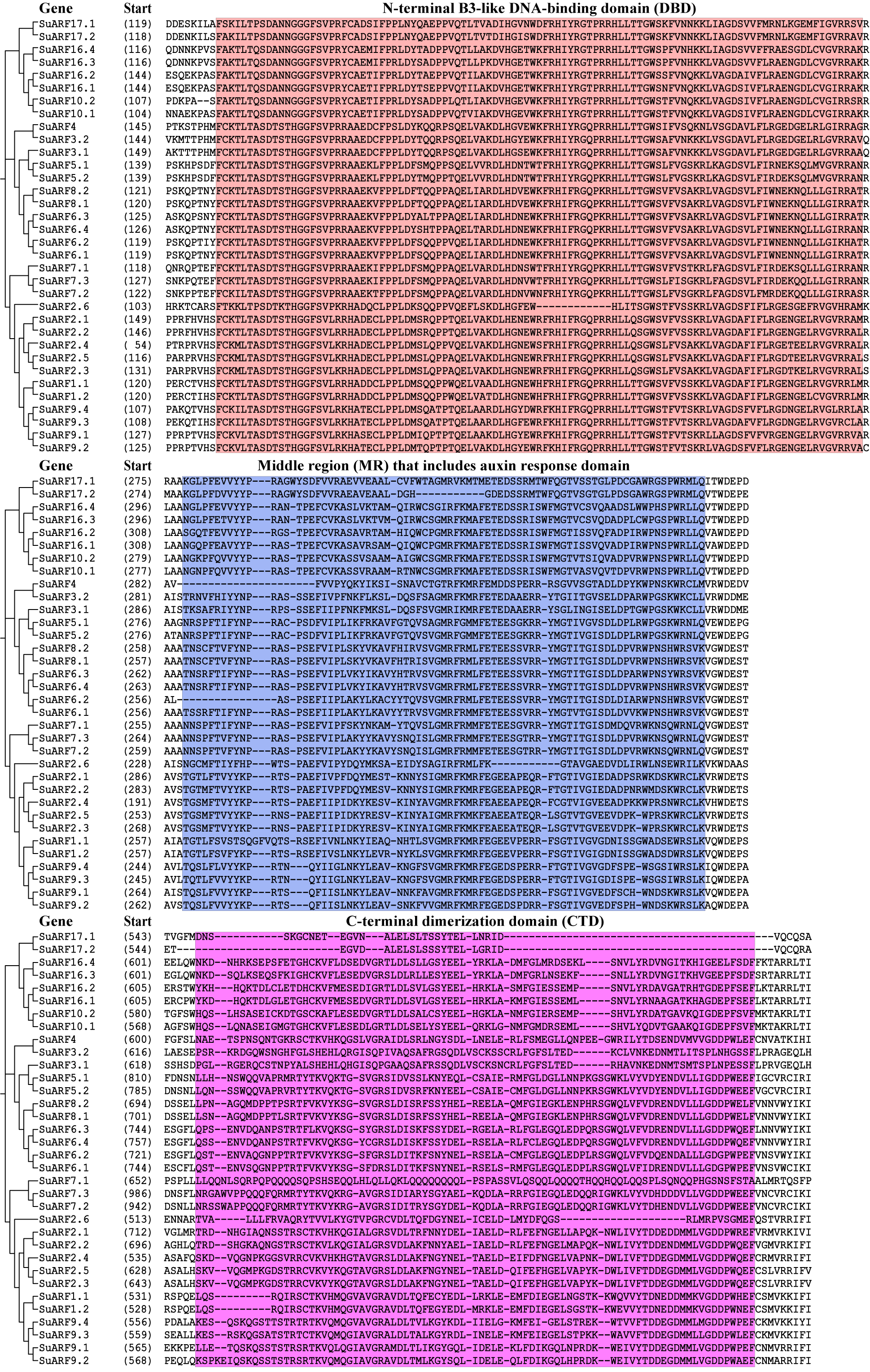


**Figure S3.** Alignment profile of conserved domains among the SuARF proteins. The shaded regions with different colors represent N-terminal B3-like DNA-binding domain (DBD), Middle region (MR) domain and C-terminal dimerization domain (CTD), respectively.


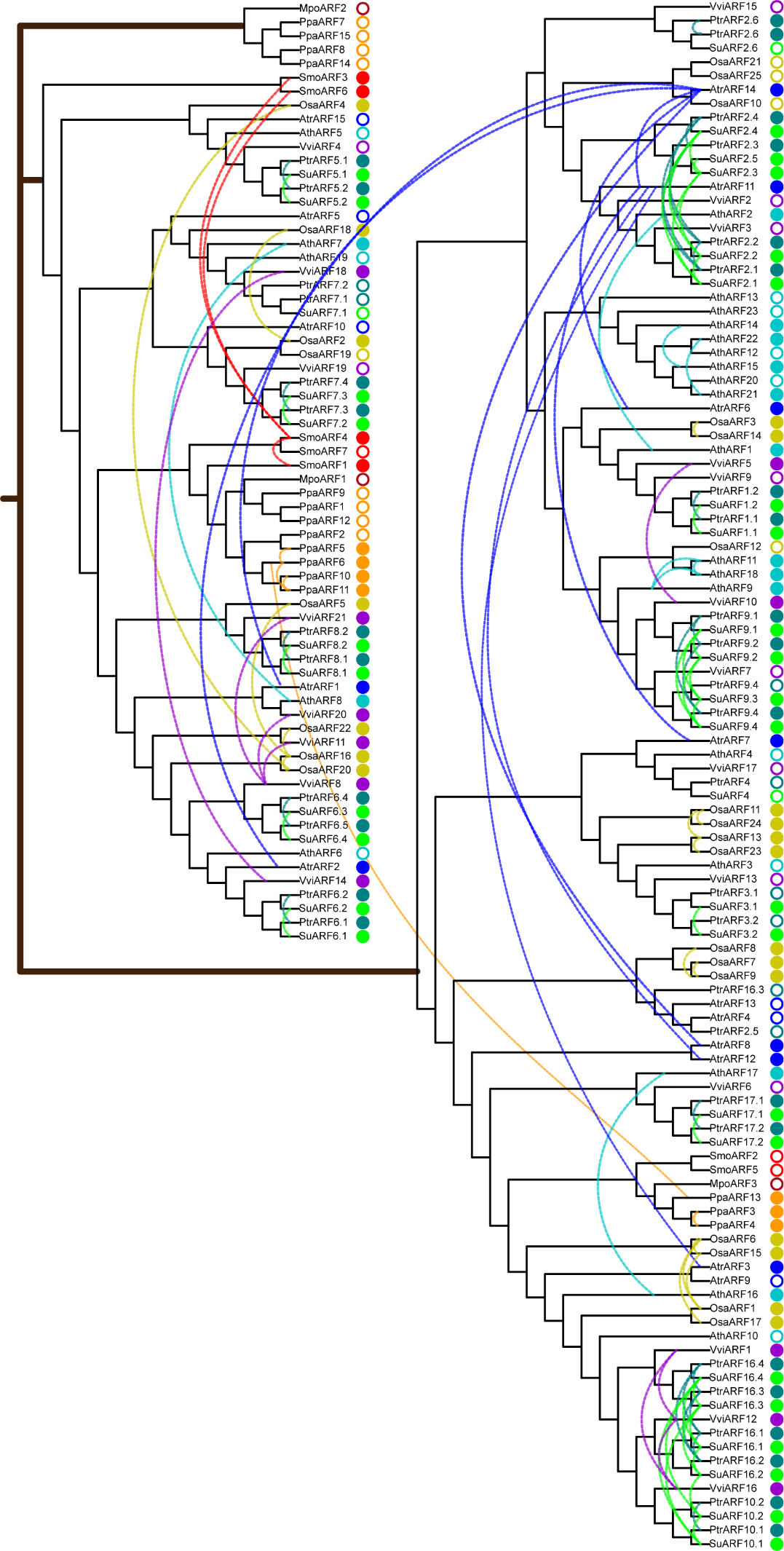


**Figure S4.** Phylogenetic relationships of ARF proteins in nine typical land plants. The neighbor-joining tree was constructed with three MpoARF, 15 PpaARF, seven SmoARF, 15 AtrARF, 25 OsaARF, 21 VviARF, 37 PtrARF, 34 SuARF, and 23 AthARF proteins. The colored solid circles indicate duplicated genes in different species, and the colored rings indicate non-duplicated genes in different species. The colored dashed links represent paralog duplicated pairs. Mpo *M. polymorpha*, Ppa *P. patens*, Smo *S. moellendorffii*, Atr *A. trichoposa*, Osa *O. sativia*, Vvi *V. vinifera*, Ptr *P. trichocarpa*, Su *S. suchowensis*, Ath *A. thaliana*.


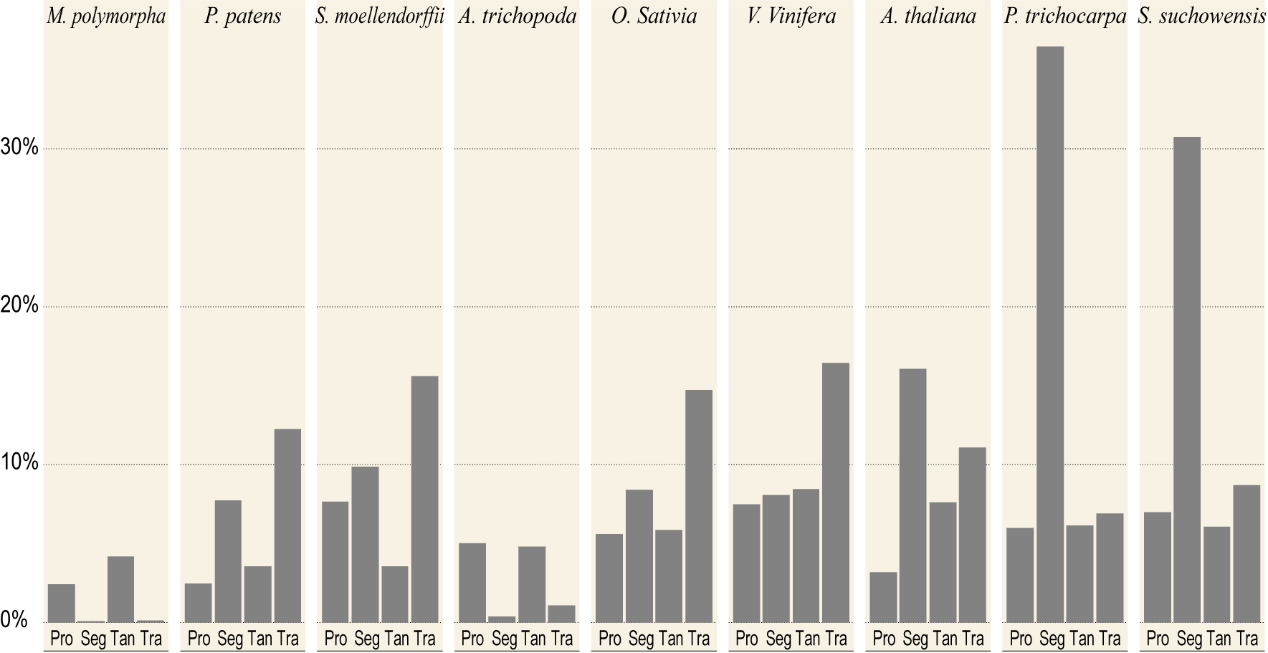


**Figure S5.** The proportion of duplicated genes in the genomes of nine species. Pro proximal duplicated genes; Seg segmental duplicated genes; Tan tandem duplicated genes; Tra transposed duplicated genes.


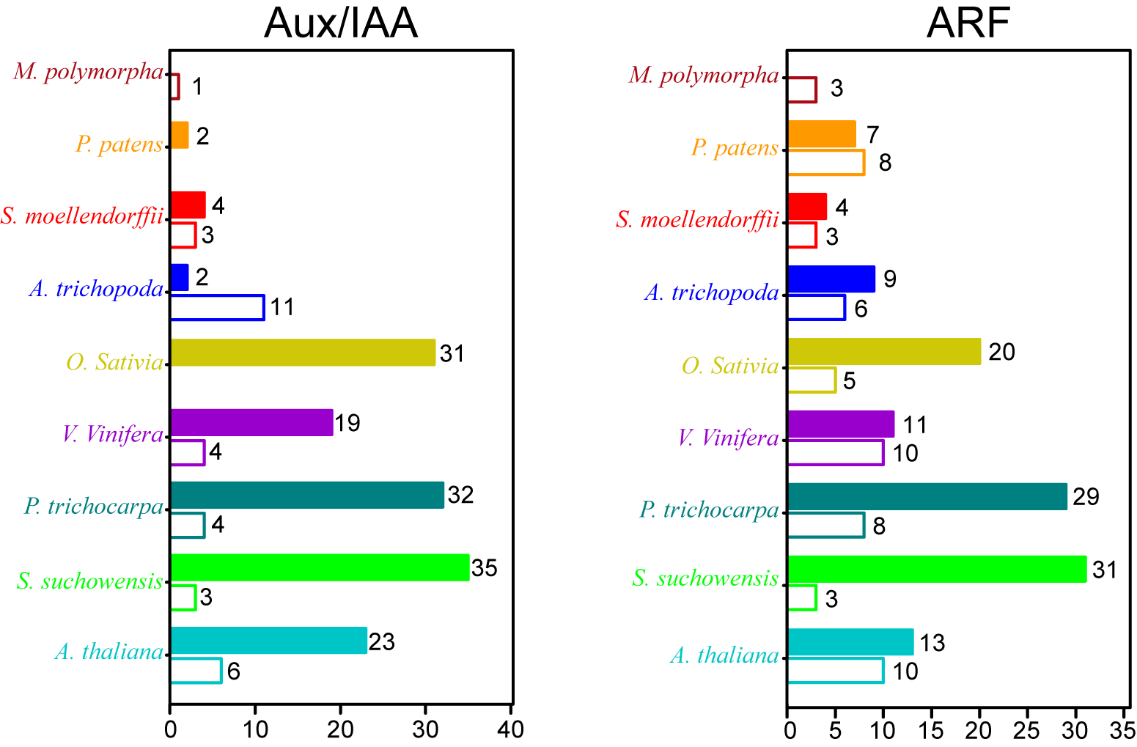


**Figure S6.** Number of duplicated genes and non-duplicated genes of Aux/IAA and ARF in nine typical land plants. The colored bars indicate the number of duplicated genes in each species, and the colored rectangles indicate the number of non-duplicated genes in each species.


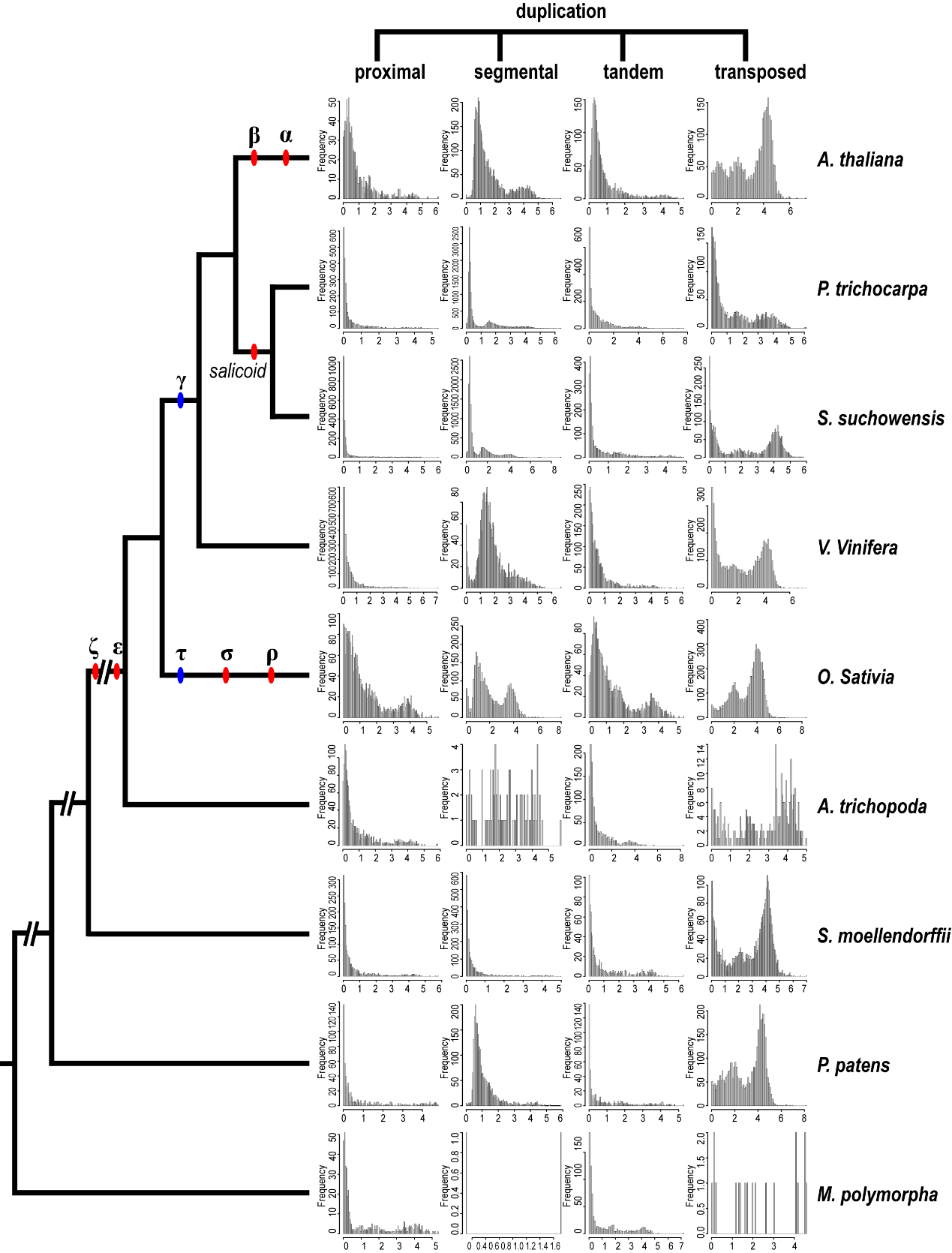


**Figure S7.** The Ks values for segmental, tandem, proximal and transposed duplicated genes in the genomes of nine species.


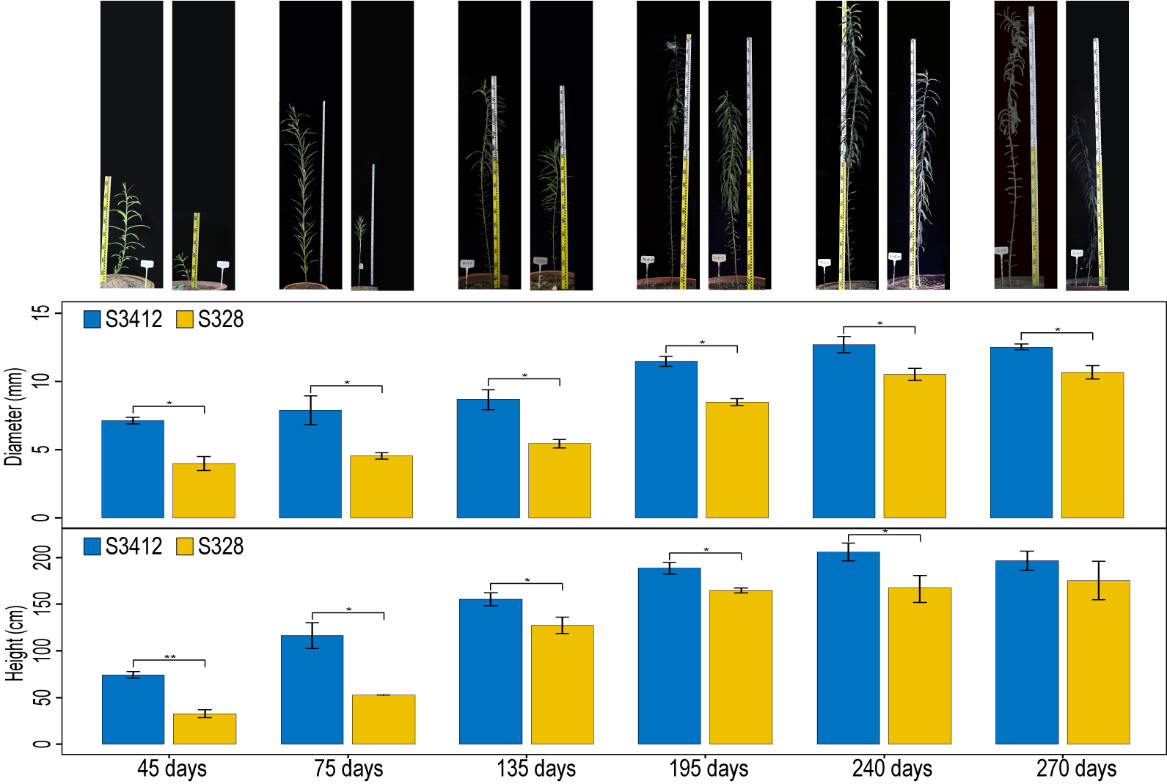


**Figure S8.** Variations in the ground diameter and stem height of the two contrasting clones “S328” and “S3412” at six growth times. Values are the mean ± SD of three replicates. Letter * represented significant difference at P < 0.05 while ** represented highly significant difference at P < 0.01 through t-test.


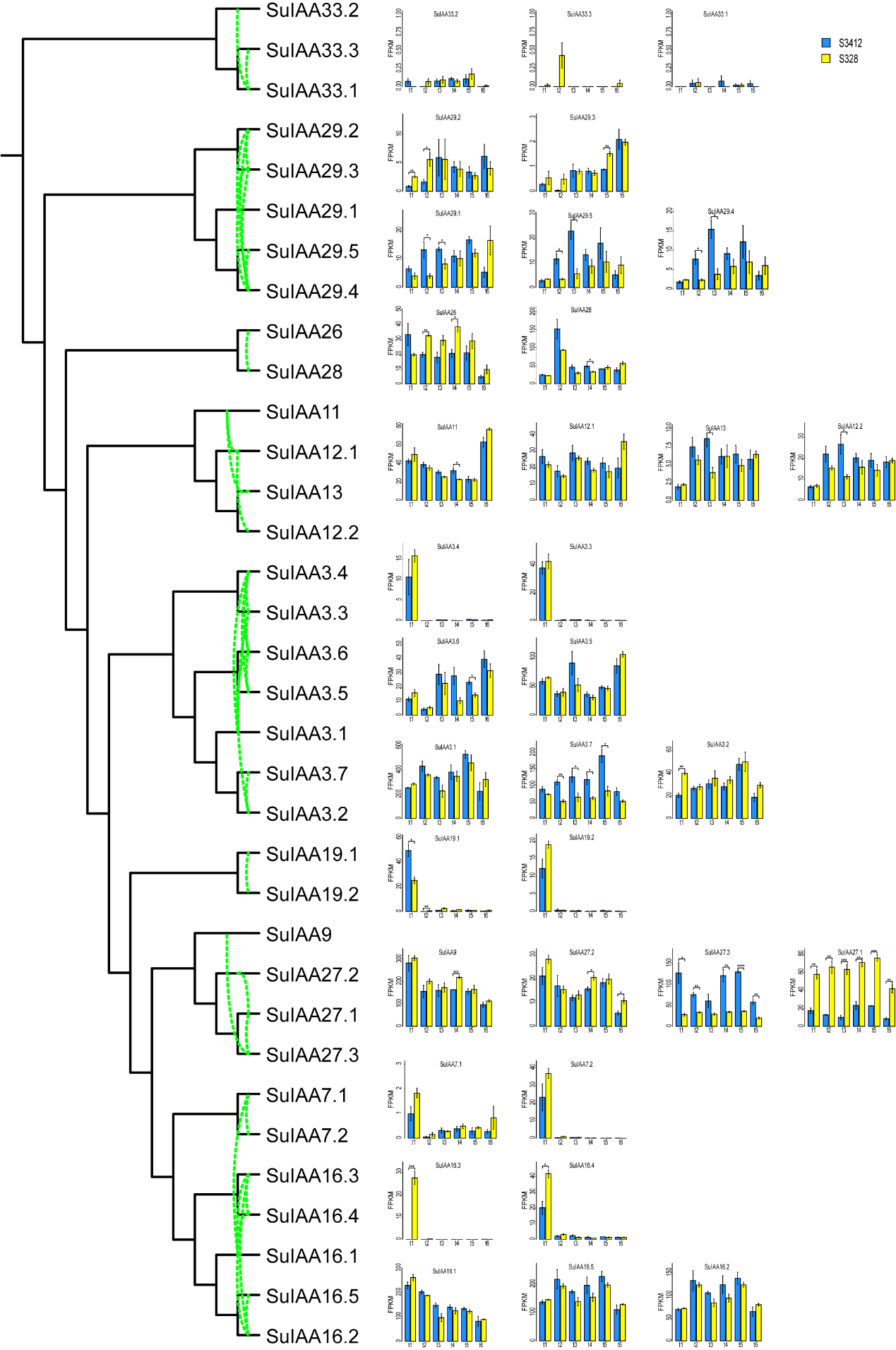


**Figure S9.** Phylogenetic tree and time-sequential transcriptome profilings of duplicated SuIAAs. The colored dashed links in tree represent paralogs duplicated pairs of SuIAAs. Each histogram shows time-sequential transcriptome profilings of each duplicated SuIAA in two contrasting clones “S328” and “S3412”. The X-axis showed the different sampling time-points (t1: 45 days after planting; t2: 75 days after planting; t3: 135 days after planting; t4: 195 days after planting; t5: 240 days after planting; t6: 270 days after planting). The Y-axis represented the FPKM value, which are the mean ± SD of three replicates, and the P value are shown as * P < 0.05, ** P < 0.01, *** P < 0.001, **** P < 0.0001.


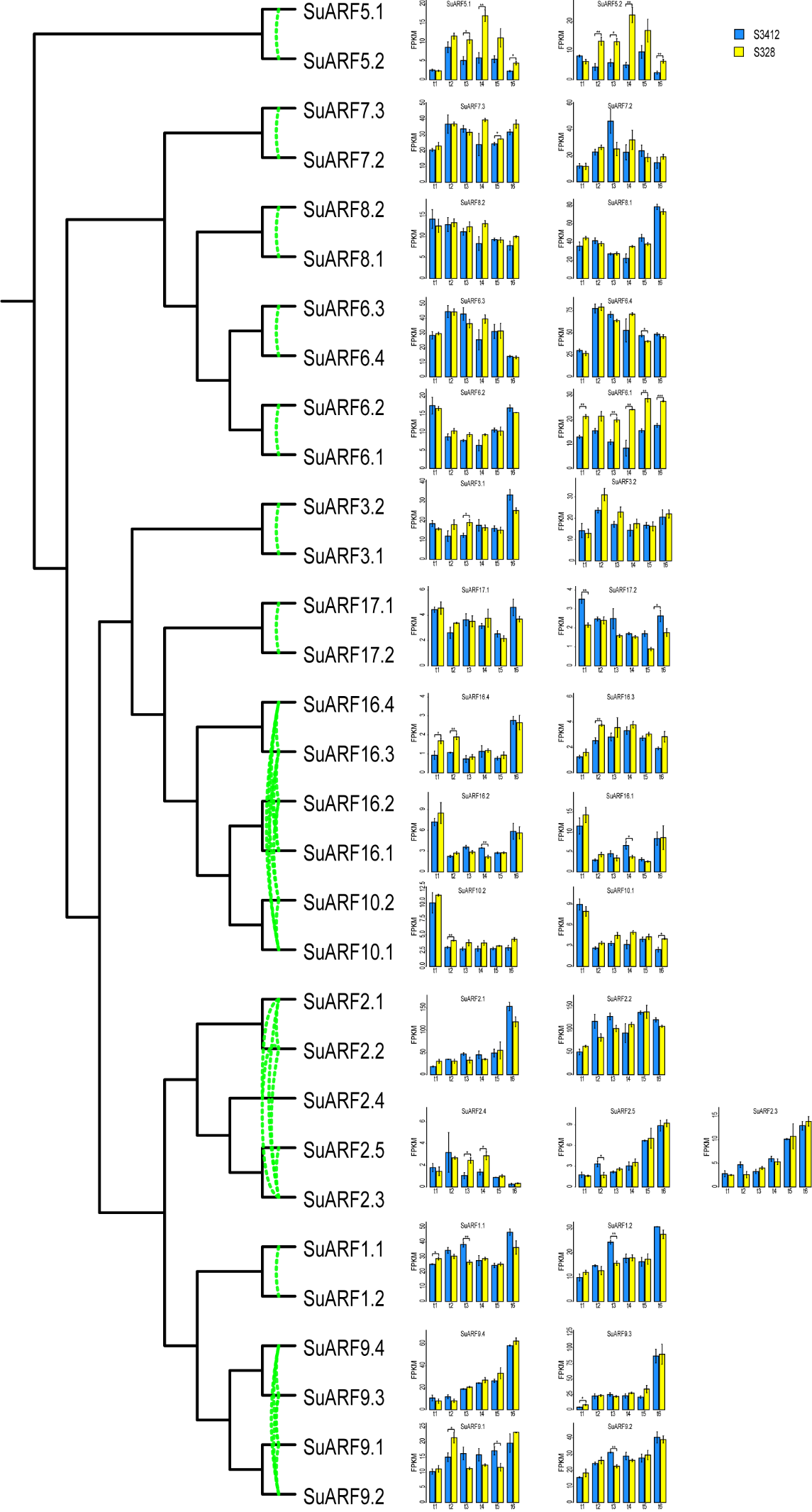


**Figure S10.** Phylogenetic tree and time-sequential transcriptome profilings of duplicated SuARFs. The colored dashed links in tree represent paralogs duplicated pairs of SuARFs. Each histogram shows time-sequential transcriptome profilings of each duplicated SuARF in two contrasting clones “S328” and “S3412”. The X-axis showed the different sampling time-points (t1: 45 days after planting; t2: 75 days after planting; t3: 135 days after planting; t4: 195 days after planting; t5: 240 days after planting; t6: 270 days after planting). The Y-axis represented the FPKM value, which are the mean ± SD of three replicates, and the P value are shown as * P < 0.05, ** P < 0.01, *** P < 0.001.


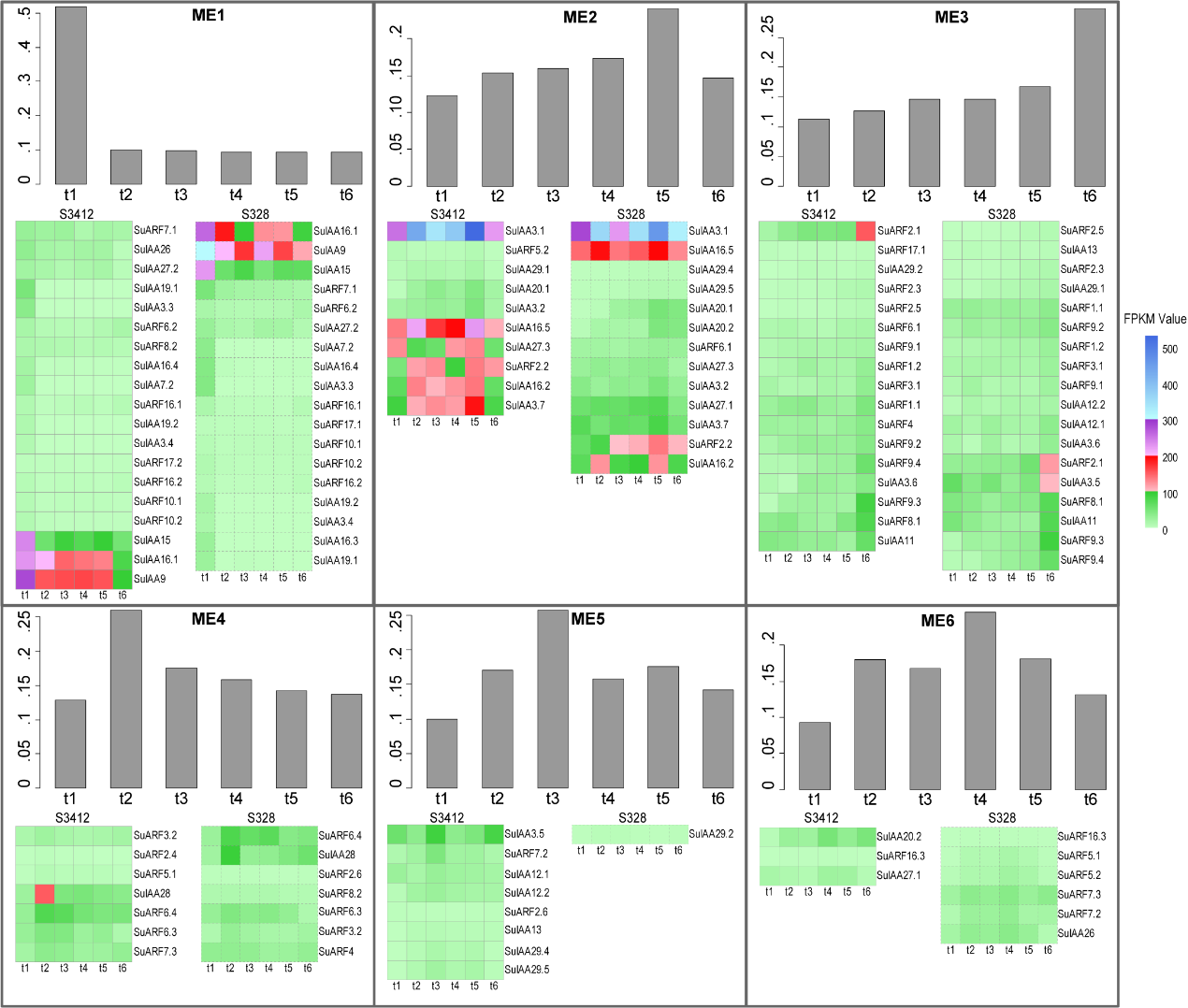


**Figure S11.** The expression patterns of the SuIAA and SuARF genes during plant growth. The change patterns of genes in each module were estimated with module eigengene (ME) values and displayed with histograms, in which six gray bars in each module represented normalized FPKM value, and the six digits represented different sampling time-points (t1: 45 days after planting; t2: 75 days after planting; t3: 135 days after planting; t4: 195 days after planting; t5: 240 days after planting; t6: 270 days after planting). The heatmap showed FPKM expression of SuIAA and SuARF genes during different growth stages, in which different colors represent the range of transcriptome expression.


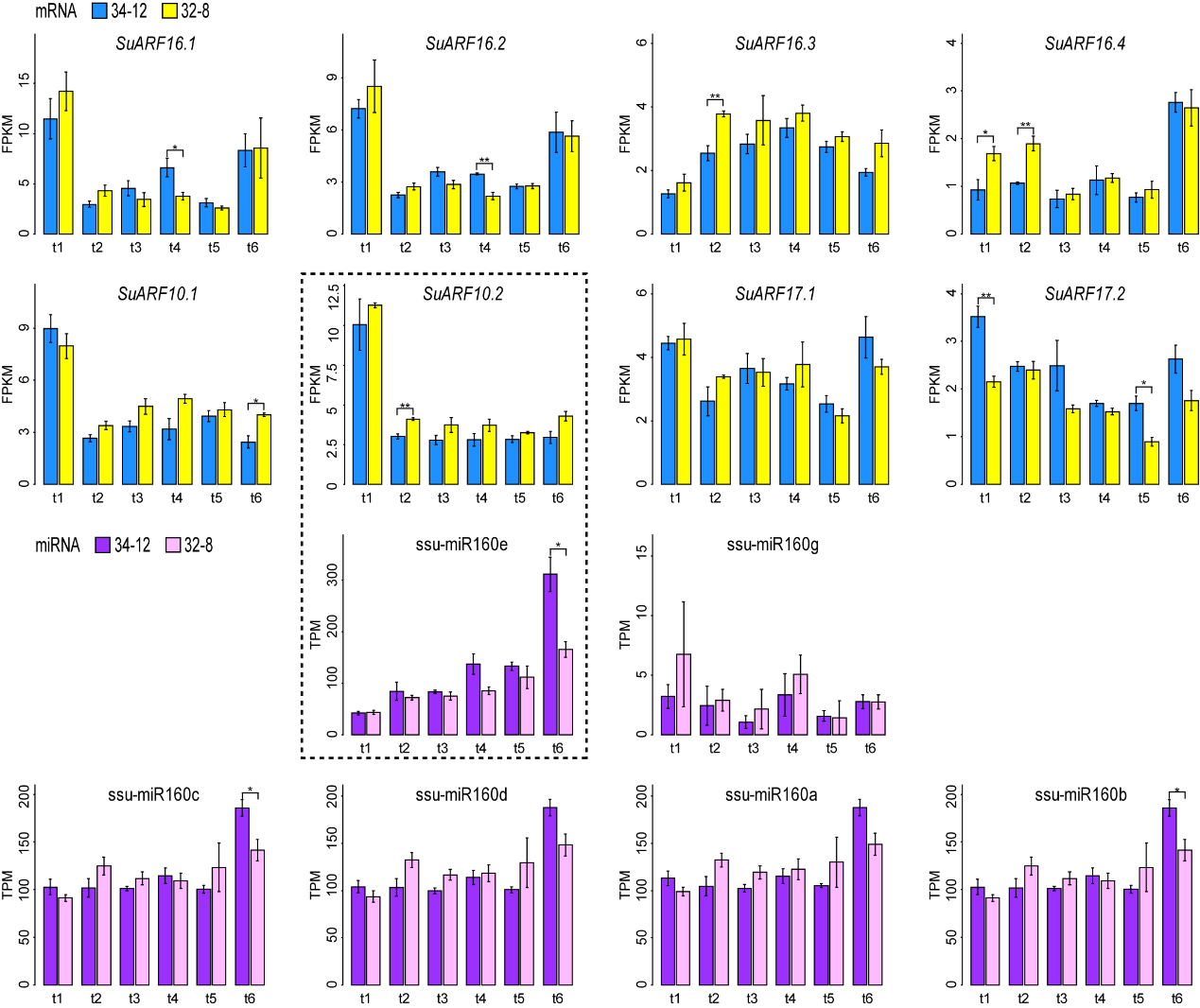


**Figure S12.** The time-sequential expression profilings of ssu-miR160s and their ARF targets.


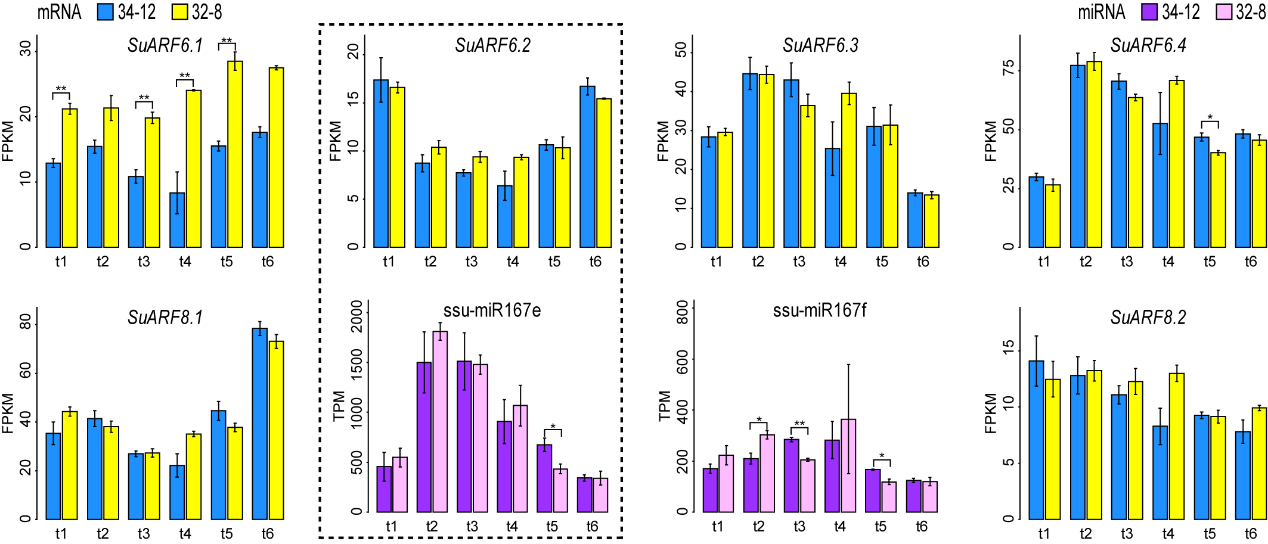


**Figure S13.** The time-sequential expression profilings of ssu-miR167s and their ARF targets.


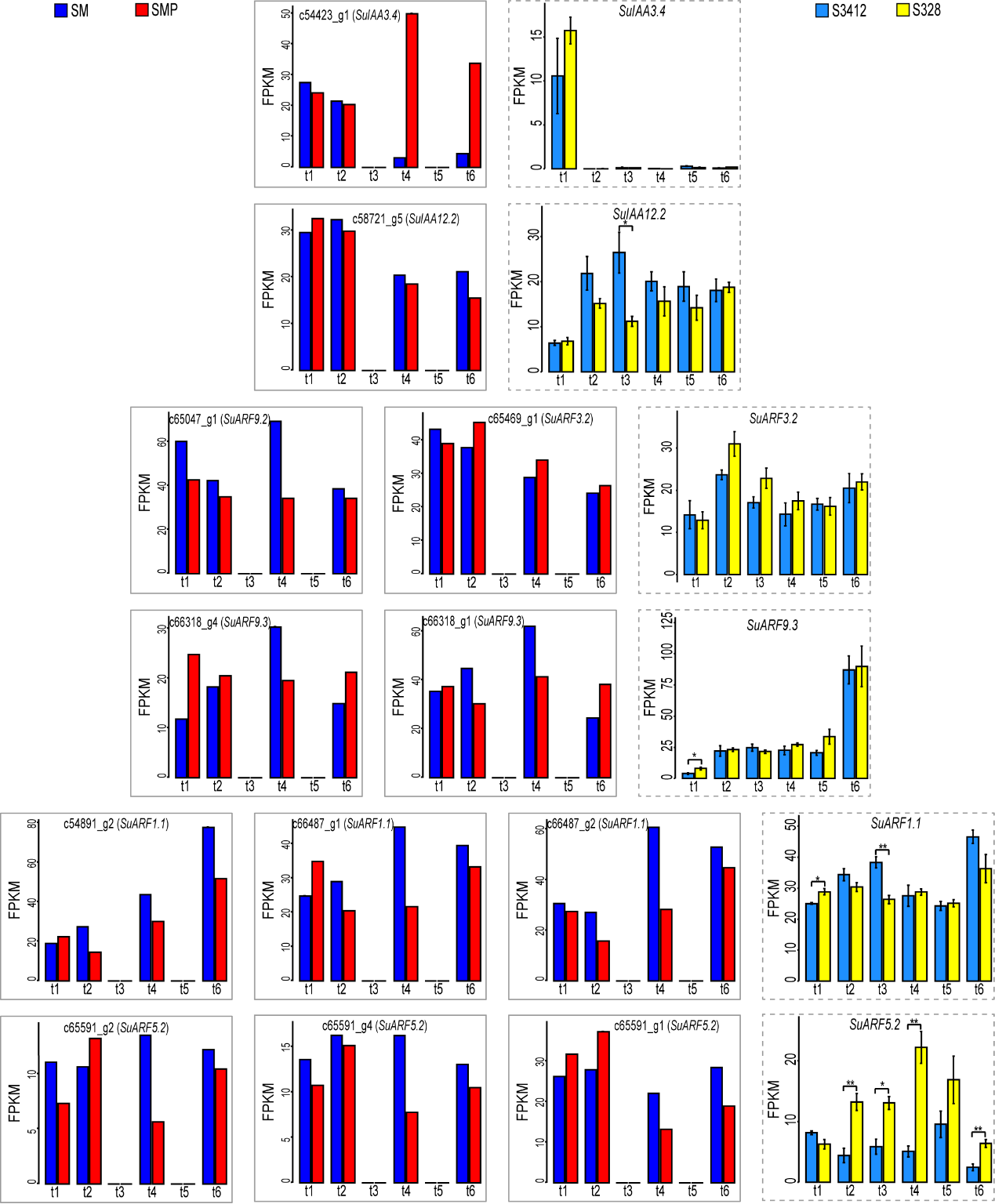


**Figure S14.** The time-sequential expression profilings of differently expressed AUX/IAA and ARF genes in *Salix matsudana* and their homogeneous genes in *Salix suchowensis*.

**Supplementary Tables**

**Table S1.** Characteristics of Aux/IAA genes family in the *Salix suchowensis* genome v2.0

| Gene Name | Chromosome | Start | End | Protein Length | Conserved Domains |
| --- | --- | --- | --- | --- | --- |
| *SuIAA11* | Chr2 | 18809276 | 18811925 | 276 | I, II, III, IV |
| *SuIAA16.4* | Chr2 | 2769230 | 2772469 | 238 | I, II, III, IV |
| *SuIAA20.1* | Chr2 | 14593913 | 14594886 | 174 | I, II, III, IV |
| *SuIAA3.3* | Chr2 | 2783080 | 2784686 | 201 | I, II, III, IV |
| *SuIAA9* | Chr2 | 7289305 | 7294970 | 365 | I, II, III, IV |
| *SuIAA19.2* | Chr3 | 3442322 | 3446453 | 185 | I, II, III, IV |
| *SuIAA27.2* | Chr3 | 2725601 | 2728884 | 343 | I, II, III, IV |
| *SuIAA16.1* | Chr5 | 3164827 | 3167873 | 247 | I, II, III, IV |
| *SuIAA16.3* | Chr5 | 16248142 | 16251159 | 231 | I, II, III, IV |
| *SuIAA3.1* | Chr5 | 3155171 | 3156204 | 190 | I, II, III, IV |
| *SuIAA3.4* | Chr5 | 16238089 | 16239843 | 199 | I, II, III, IV |
| *SuIAA28* | Chr6 | 17678073 | 17681583 | 200 | II, III, IV |
| *SuIAA29.1* | Chr6 | 18848750 | 18851446 | 231 | II, III, IV |
| *SuIAA29.3* | Chr6 | 4011742 | 4012767 | 231 | II, III, IV |
| *SuIAA33.2* | Chr6 | 12154904 | 12156837 | 169 | III, IV |
| *SuIAA12.2* | Chr8 | 9833689 | 9837165 | 303 | I, II, III, IV |
| *SuIAA13* | Chr8 | 9718353 | 9722594 | 296 | I, II, III, IV |
| *SuIAA12.1* | Chr10 | 3401033 | 3403914 | 310 | I, II, III, IV |
| *SuIAA3.6* | Chr10 | 5680397 | 5682085 | 197 | I, II, III, IV |
| *SuIAA34* | Chr10 | 8413834 | 8415585 | 136 | III, IV |
| *SuIAA7.1* | Chr10 | 5716557 | 5718647 | 240 | I, II, III, IV |
| *SuIAA16.2* | Chr13 | 2906150 | 2908262 | 246 | I, II, III, IV |
| *SuIAA16.5* | Chr13 | 2950704 | 2953539 | 246 | I, II, III, IV |
| *SuIAA3.2* | Chr13 | 2918575 | 2920226 | 204 | I, II, III, IV |
| *SuIAA3.7* | Chr13 | 2939415 | 2940518 | 204 | I, II, III, IV |
| *SuIAA20.2* | Chr14 | 7060623 | 7061553 | 176 | I, III, IV |
| *SuIAA15* | Chr16 | 13951261 | 13953696 | 220 | I, II, III, IV |
| *SuIAA19.1* | Chr16 | 13943495 | 13947088 | 194 | I, II, III, IV |
| *SuIAA26* | Chr16 | 14220403 | 14224441 | 340 | I, II, III, IV |
| *SuIAA27.1* | Chr16 | 14501244 | 14504506 | 346 | I, II, III, IV |
| *SuIAA27.3* | Chr16 | 14555673 | 14558436 | 326 | I, II, III, IV |
| *SuIAA29.2* | Chr18 | 11754104 | 11755509 | 234 | II, III, IV |
| *SuIAA29.4* | Chr18 | 2066564 | 2067957 | 227 | II, III, IV |
| *SuIAA29.5* | Chr18 | 2147853 | 2148900 | 227 | II, III, IV |
| *SuIAA33.1* | Chr18 | 9204456 | 9205637 | 174 | III, IV |
| *SuIAA33.3* | Chr18 | 9238589 | 9239902 | 174 | III, IV |
| *SuIAA3.5* | Contig00694 | 162916 | 164917 | 194 | I, II, III, IV |
| *SuIAA7.2* | Contig00694 | 178526 | 181047 | 250 | I, II, III, IV |

**Table S2.** Characteristics of ARF genes family in the *Salix suchowensis* genome v2.0

| Gene Name | Chromosome | Start | End | Protein Length | Conserved Domains |
| --- | --- | --- | --- | --- | --- |
| *SuARF16.3* | Chr1 | 9603011 | 9607173 | 712 | DBD, MR |
| *SuARF6.1* | Chr1 | 25250829 | 25258779 | 906 | DBD, MR, CTD |
| *SuARF17.1* | Chr2 | 5914597 | 5919700 | 593 | DBD, MR |
| *SuARF5.1* | Chr2 | 1491797 | 1498076 | 942 | DBD, MR, CTD |
| *SuARF6.3* | Chr2 | 3458978 | 3467400 | 925 | DBD, MR, CTD |
| *SuARF9.3* | Chr2 | 13643406 | 13647923 | 692 | DBD, MR, CTD |
| *SuARF1.1* | Chr3 | 56472 | 61864 | 668 | DBD, MR, CTD |
| *SuARF2.4* | Chr3 | 11214039 | 11219068 | 677 | DBD, MR, CTD |
| *SuARF9.1* | Chr3 | 9946266 | 9950346 | 695 | DBD, MR, CTD |
| *SuARF10.1* | Chr4 | 16073742 | 16078144 | 700 | DBD, MR, CTD |
| *SuARF3.1* | Chr4 | 2696603 | 2703240 | 741 | DBD, MR |
| *SuARF8.1* | Chr4 | 4334989 | 4344299 | 832 | DBD, MR, CTD |
| *SuARF17.2* | Chr5 | 12147208 | 12150080 | 581 | DBD, MR |
| *SuARF5.2* | Chr5 | 17637399 | 17642409 | 914 | DBD, MR, CTD |
| *SuARF6.4* | Chr5 | 15406241 | 15414470 | 913 | DBD, MR, CTD |
| *SuARF16.4* | Chr6 | 8731536 | 8735554 | 706 | DBD, MR |
| *SuARF7.1* | Chr6 | 11189494 | 11197322 | 807 | DBD, MR |
| *SuARF7.3* | Chr6 | 4794509 | 4801994 | 1117 | DBD, MR |
| *SuARF16.2* | Chr8 | 1948787 | 1952426 | 708 | DBD, MR |
| *SuARF10.2* | Chr9 | 812342 | 816043 | 693 | DBD, MR |
| *SuARF4* | Chr9 | 1103740 | 1110412 | 746 | DBD, MR, CTD |
| *SuARF16.1* | Chr10 | 15132192 | 15135879 | 708 | DBD, MR |
| *SuARF3.2* | Chr11 | 3799294 | 3806182 | 747 | DBD, MR |
| *SuARF6.2* | Chr11 | 9689639 | 9695677 | 868 | DBD, MR, CTD |
| *SuARF2.1* | Chr12 | 8444211 | 8450171 | 856 | DBD, MR |
| *SuARF2.6* | Chr14 | 8469314 | 8475677 | 608 | DBD, MR |
| *SuARF9.4* | Chr14 | 6114500 | 6119383 | 689 | DBD, MR, CTD |
| *SuARF2.2* | Chr15 | 10663901 | 10668177 | 842 | DBD, MR, CTD |
| *SuARF1.2* | Chr16 | 16174273 | 16181103 | 667 | DBD, MR, CTD |
| *SuARF2.3* | Chr16 | 3798407 | 3803839 | 785 | DBD, MR, CTD |
| *SuARF2.5* | Chr16 | 3832851 | 3838849 | 770 | DBD, MR, CTD |
| *SuARF9.2* | Chr16 | 5349356 | 5353933 | 700 | DBD, MR, CTD |
| *SuARF8.2* | Chr17 | 13163854 | 13172328 | 821 | DBD, MR, CTD |
| *SuARF7.2* | Chr18 | 13189520 | 13196553 | 1074 | DBD, MR, CTD |

**Table S3.** The statistics of duplicated genes in the genomes of nine species

| Species | Genome Version | Genes Number | Proximal duplicated Genes (%) | Segmental duplicated genes (%) | tandem duplicated genes (%) | transposed duplicated genes (%) | Total duplicated genes (%) |
| --- | --- | --- | --- | --- | --- | --- | --- |
| *M. polymorpha* | v3.1 | 19,287 | 468  (2.43%) | 17  (0.09%) | 808  (4.19%) | 21  (0.11%) | 1314  (6.82%) |
| *P. patens* | v3.3 | 32,926 | 811  (2.46%) | 2552  (7.75%) | 1173  (3.56%) | 4036  (12.26%) | 8572  (26.03%) |
| *S. moellendorffii* | v1.0 | 22,285 | 1710  (7.67%) | 2205  (9.89%) | 792  (3.55%) | 3482  (15.62%) | 8189  (36.73%) |
| *A. trichoposa* | v1.0 | 26,846 | 1344  (5.01%) | 95  (0.35%) | 1292  (4.81%) | 288  (1.07%) | 3019  (11.24%) |
| *O. sativia* | v7.0 | 42,189 | 2371  (5.62%) | 3549  (8.41%) | 2466  (5.85%) | 6218  (14.74%) | 14604  (34.62%) |
| *V. vinifera* | v2.1 | 31,845 | 2388  (7.5%) | 2574  (8.08%) | 2687  (8.44%) | 5231  (16.43%) | 12880  (40.45%) |
| *A. thaliana* | v11 | 27,654 | 877  (3.17%) | 4439  (16.05%) | 2104  (7.61%) | 3069  (11.1%) | 10489  (37.93%) |
| *P. trichocarpa* | v3.1 | 42,950 | 2558  (5.96%) | 15,677  (36.5%) | 2643  (6.15%) | 2967  (6.91%) | 23845  (55.52%) |
| *S. suchowensis* | v2.0 | 36,937 | 2574  (6.97%) | 11,369  (30.78%) | 2232  (6.04%) | 3212  (8.7%) | 19387  (52.49%) |

**Table S4.** The statistics of Ka, Ks, and Ka/Ks values for segmental, tandem, proximal and transposed duplicated Aux/IAA genes in the genomes of *A. thaliana*, *P. trichocarpa*, *S. suchowensis*, *V. vinifera*, *O. sativia*, *A. trichoposa*, *S. moellendorffii*, and *P. patens*.

| Duplicated1 | Location 1 | Duplicated2 | Location 2 | Ka | Ks | Ka/Ks | Mode |
| --- | --- | --- | --- | --- | --- | --- | --- |
| *AthIAA10* | Chr1:1059470 | *AthIAA11* | Chr4:14142135 | 0.36 | 2.31 | 0.16 | segmental |
| *AthIAA3* | Chr1:1128149 | *AthIAA2* | Chr3:8180646 | 0.43 | 3.78 | 0.12 | segmental |
| *AthIAA3* | Chr1:1128149 | *AthIAA1* | Chr4:8360996 | 0.25 | 3.47 | 0.07 | segmental |
| *AthIAA3* | Chr1:1128149 | *AthIAA4* | Chr5:17550179 | 0.15 | 0.85 | 0.17 | segmental |
| *AthIAA17* | Chr1:1136078 | *AthIAA7* | Chr3:8194606 | 0.19 | 3.59 | 0.05 | segmental |
| *AthIAA17* | Chr1:1136078 | *AthIAA14* | Chr4:8348579 | 0.23 | 3.69 | 0.06 | segmental |
| *AthIAA12* | Chr1:1240294 | *AthIAA13* | Chr2:14114281 | 0.13 | 1.11 | 0.12 | segmental |
| *AthIAA34* | Chr1:5181991 | *AthIAA32* | Chr2:118018 | 0.2 | 0.56 | 0.35 | segmental |
| *AthIAA5* | Chr1:5365512 | *AthIAA19* | Chr3:5264001 | 0.29 | 3.53 | 0.08 | segmental |
| *AthIAA18* | Chr1:19305081 | *AthIAA26* | Chr3:5612203 | 0.18 | 1.14 | 0.16 | segmental |
| *AthIAA6* | Chr1:19672476 | *AthIAA19* | Chr3:5264001 | 0.23 | 0.95 | 0.24 | segmental |
| *AthIAA8* | Chr2:9636380 | *AthIAA9* | Chr5:26253408 | 0.31 | 2.37 | 0.13 | segmental |
| *AthIAA20* | Chr2:19307714 | *AthIAA30* | Chr3:22995686 | 0.09 | 0.98 | 0.09 | segmental |
| *AthIAA2* | Chr3:8180646 | *AthIAA14* | Chr4:8348579 | 0.36 | 3.52 | 0.1 | segmental |
| *AthIAA2* | Chr3:8180646 | *AthIAA4* | Chr5:17550179 | 0.21 | 3.5 | 0.06 | segmental |
| *AthIAA14* | Chr4:8348579 | *AthIAA1* | Chr4:8360996 | 0.36 | 3.49 | 0.1 | tandem |
| *AthIAA27* | Chr4:14323296 | *AthIAA9* | Chr5:26253408 | 0.35 | 3.95 | 0.09 | transposed |
| *PtrIAA19.1* | Chr01:15200826 | *PtrIAA19.3* | Chr03:8438848 | 0.07 | 0.17 | 0.39 | segmental |
| *PtrIAA27.3* | Chr01:16403405 | *PtrIAA27.2* | Chr03:7543098 | 0.06 | 0.16 | 0.36 | segmental |
| *PtrIAA26.1* | Chr01:17118183 | *PtrIAA26.1* | Chr03:7029803 | 0.06 | 0.26 | 0.22 | segmental |
| *PtrIAA26.1* | Chr01:17118183 | *PtrIAA28.2* | Chr06:24541267 | 0.43 | 3.91 | 0.11 | segmental |
| *PtrIAA16.4* | Chr02:2855340 | *PtrIAA16.1* | Chr05:3853836 | 0.18 | 2.01 | 0.09 | segmental |
| *PtrIAA16.4* | Chr02:2855340 | *PtrIAA16.3* | Chr05:23058432 | 0.03 | 0.28 | 0.11 | segmental |
| *PtrIAA16.4* | Chr02:2855340 | *PtrIAA7.2* | Chr08:10964563 | 0.25 | 1.68 | 0.15 | segmental |
| *PtrIAA16.4* | Chr02:2855340 | *PtrIAA7.1* | Chr10:10326360 | 0.21 | 1.57 | 0.14 | segmental |
| *PtrIAA16.4* | Chr02:2855340 | *PtrIAA16.2* | Chr13:2911420 | 0.2 | 2.53 | 0.08 | segmental |
| *PtrIAA3.3* | Chr02:2867260 | *PtrIAA3.1* | Chr05:3843828 | 0.23 | 3.65 | 0.06 | segmental |
| *PtrIAA3.3* | Chr02:2867260 | *PtrIAA3.4* | Chr05:23047878 | 0.03 | 0.19 | 0.16 | segmental |
| *PtrIAA3.3* | Chr02:2867260 | *PtrIAA3.5* | Chr08:10949306 | 0.22 | 2.66 | 0.08 | segmental |
| *PtrIAA3.3* | Chr02:2867260 | *PtrIAA3.6* | Chr10:10350320 | 0.22 | 1.59 | 0.14 | segmental |
| *PtrIAA3.3* | Chr02:2867260 | *PtrIAA3.2* | Chr13:2893391 | 0.2 | 2.6 | 0.08 | segmental |
| *PtrIAA20.1* | Chr02:14613581 | *PtrIAA20.2* | Chr14:8739749 | 0.08 | 0.25 | 0.32 | segmental |
| *PtrIAA11* | Chr02:24564672 | *PtrIAA12.2* | Chr08:11761172 | 0.36 | 2.35 | 0.16 | segmental |
| *PtrIAA11* | Chr02:24564672 | *PtrIAA12.1* | Chr10:9283230 | 0.41 | 2.28 | 0.18 | segmental |
| *PtrIAA26.1* | Chr03:7029803 | *PtrIAA28.2* | Chr06:24541267 | 0.48 | 1.72 | 0.28 | segmental |
| *PtrIAA3.1* | Chr05:3843828 | *PtrIAA3.4* | Chr05:23047878 | 0.22 | 3.7 | 0.06 | segmental |
| *PtrIAA3.1* | Chr05:3843828 | *PtrIAA3.6* | Chr10:10350320 | 0.22 | 1.54 | 0.15 | segmental |
| *PtrIAA3.1* | Chr05:3843828 | *PtrIAA3.2* | Chr13:2893391 | 0.06 | 0.29 | 0.21 | segmental |
| *PtrIAA16.1* | Chr05:3853836 | *PtrIAA16.3* | Chr05:23058432 | 0.18 | 1.95 | 0.09 | segmental |
| *PtrIAA16.1* | Chr05:3853836 | *PtrIAA7.2* | Chr08:10964563 | 0.18 | 1.36 | 0.13 | segmental |
| *PtrIAA16.1* | Chr05:3853836 | *PtrIAA7.1* | Chr10:10326360 | 0.18 | 1.31 | 0.14 | segmental |
| *PtrIAA16.1* | Chr05:3853836 | *PtrIAA16.2* | Chr13:2911420 | 0.06 | 0.31 | 0.21 | segmental |
| *PtrIAA3.4* | Chr05:23047878 | *PtrIAA3.5* | Chr08:10949306 | 0.21 | 1.76 | 0.12 | segmental |
| *PtrIAA3.4* | Chr05:23047878 | *PtrIAA3.6* | Chr10:10350320 | 0.19 | 2.42 | 0.08 | segmental |
| *PtrIAA3.4* | Chr05:23047878 | *PtrIAA3.2* | Chr13:2893391 | 0.2 | 3.62 | 0.06 | segmental |
| *PtrIAA16.3* | Chr05:23058432 | *PtrIAA7.2* | Chr08:10964563 | 0.22 | 1.25 | 0.18 | segmental |
| *PtrIAA16.3* | Chr05:23058432 | *PtrIAA16.2* | Chr13:2911420 | 0.17 | 1.72 | 0.1 | segmental |
| *PtrIAA29.3* | Chr06:4973190 | *PtrIAA29.1* | Chr06:26002972 | 0.45 | 2.23 | 0.2 | segmental |
| *PtrIAA29.3* | Chr06:4973190 | *PtrIAA29.2* | Chr18:15004734 | 0.07 | 0.2 | 0.35 | segmental |
| *PtrIAA33.1* | Chr06:17150336 | *PtrIAA33.1* | Chr18:12176424 | 0.1 | 0.25 | 0.41 | segmental |
| *PtrIAA28.2* | Chr06:24541267 | *PtrIAA28.2* | Chr18:5997995 | 0.1 | 0.21 | 0.5 | segmental |
| *PtrIAA29.1* | Chr06:26002972 | *PtrIAA29.2* | Chr18:15004734 | 0.44 | 1.38 | 0.32 | segmental |
| *PtrIAA3.5* | Chr08:10949306 | *PtrIAA3.6* | Chr10:10350320 | 0.04 | 0.28 | 0.14 | segmental |
| *PtrIAA3.5* | Chr08:10949306 | *PtrIAA3.2* | Chr13:2893391 | 0.2 | 3.63 | 0.05 | segmental |
| *PtrIAA7.2* | Chr08:10964563 | *PtrIAA7.1* | Chr10:10326360 | 0.02 | 0.13 | 0.19 | segmental |
| *PtrIAA7.2* | Chr08:10964563 | *PtrIAA16.2* | Chr13:2911420 | 0.19 | 2.82 | 0.07 | segmental |
| *PtrIAA12.2* | Chr08:11761172 | *PtrIAA12.1* | Chr10:9283230 | 0.13 | 0.32 | 0.41 | segmental |
| *PtrIAA7.1* | Chr10:10326360 | *PtrIAA16.2* | Chr13:2911420 | 0.2 | 1.23 | 0.16 | segmental |
| *PtrIAA3.6* | Chr10:10350320 | *PtrIAA3.2* | Chr13:2893391 | 0.2 | 1.67 | 0.12 | segmental |
| *PtrIAA27.1* | Chr06:15330357 | *PtrIAA27.1* | Chr06:15365885 | 0.12 | 0.2 | 0.6 | proximal |
| *SuIAA29.5* | chr18:2147853 | *SuIAA29.4* | chr18:2066564 | 0 | 0 | 0 | proximal |
| *SuIAA33.1* | chr18:9204456 | *SuIAA33.3* | chr18:9238589 | 0.01 | 0.01 | 0.65 | proximal |
| *SuIAA16.5* | chr13:2950704 | *SuIAA16.2* | chr13:2906150 | 0 | 0.01 | 0 | proximal |
| *SuIAA3.7* | chr13:2939415 | *SuIAA3.2* | chr13:2918575 | 0 | 0 | 0 | proximal |
| *SuIAA27.3* | chr16:14555673 | *SuIAA27.1* | chr16:14501244 | 0.01 | 0.02 | 0.29 | proximal |
| *SuIAA29.1* | chr06:18848750 | *SuIAA29.4* | chr18:2066564 | 0.13 | 0.23 | 0.58 | segmental |
| *SuIAA29.1* | chr06:18848750 | *SuIAA29.2* | chr18:11754104 | 0.44 | 1.99 | 0.22 | segmental |
| *SuIAA3.3* | chr02:2783080 | *SuIAA3.4* | chr05:16238089 | 0.08 | 0.31 | 0.25 | segmental |
| *SuIAA3.3* | chr02:2783080 | *SuIAA3.2* | chr13:2918575 | 0.22 | 3.62 | 0.06 | segmental |
| *SuIAA3.3* | chr02:2783080 | *SuIAA3.6* | chr10:5680397 | 0.22 | 1.53 | 0.14 | segmental |
| *SuIAA27.2* | chr03:2725601 | *SuIAA27.1* | chr16:14501244 | 0.07 | 0.28 | 0.23 | segmental |
| *SuIAA29.1* | chr06:18848750 | *SuIAA29.3* | chr06:4011742 | 0.5 | 1.77 | 0.28 | segmental |
| *SuIAA29.3* | chr06:4011742 | *SuIAA29.4* | chr18:2066564 | 0.49 | 1.47 | 0.34 | segmental |
| *SuIAA29.3* | chr06:4011742 | *SuIAA29.2* | chr18:11754104 | 0.11 | 0.41 | 0.27 | segmental |
| *SuIAA12.1* | chr10:3401033 | *SuIAA12.2* | chr08:9833689 | 0.08 | 0.28 | 0.3 | segmental |
| *SuIAA7.2* | Contig00694:178526 | *SuIAA7.1* | chr10:5716557 | 0.05 | 0.18 | 0.29 | segmental |
| *SuIAA16.3* | chr05:16248142 | *SuIAA16.2* | chr13:2906150 | 0.21 | 1.7 | 0.12 | segmental |
| *SuIAA29.4* | chr18:2066564 | *SuIAA29.2* | chr18:11754104 | 0.47 | 1.7 | 0.28 | segmental |
| *SuIAA19.1* | chr16:13943495 | *SuIAA19.2* | chr03:3442322 | 0.11 | 0.31 | 0.35 | segmental |
| *SuIAA3.4* | chr05:16238089 | *SuIAA3.6* | chr10:5680397 | 0.23 | 2.46 | 0.09 | segmental |
| *SuIAA16.3* | chr05:16248142 | *SuIAA16.4* | chr02:2769230 | 0.07 | 0.41 | 0.16 | segmental |
| *SuIAA16.4* | chr02:2769230 | *SuIAA16.1* | chr05:3164827 | 0.21 | 3.06 | 0.07 | segmental |
| *SuIAA28* | chr06:17678073 | *SuIAA26* | chr16:14220403 | 0.32 | 1.67 | 0.19 | segmental |
| *SuIAA16.3* | chr05:16248142 | *SuIAA16.1* | chr05:3164827 | 0.2 | 1.95 | 0.1 | segmental |
| *SuIAA16.1* | chr05:3164827 | *SuIAA16.2* | chr13:2906150 | 0.09 | 0.48 | 0.18 | segmental |
| *SuIAA16.1* | chr05:3164827 | *SuIAA7.1* | chr10:5716557 | 0.21 | 1.55 | 0.13 | segmental |
| *SuIAA3.4* | chr05:16238089 | *SuIAA3.5* | Contig00694:162916 | 0.29 | 1.98 | 0.15 | segmental |
| *SuIAA3.5* | Contig00694:162916 | *SuIAA3.6* | chr10:5680397 | 0.13 | 0.41 | 0.33 | segmental |
| *SuIAA27.3* | chr16:14555673 | *SuIAA27.1* | chr16:14501244 | 0.10 | 0.20 | 0.50 | segmental |
| *SuIAA12.1* | chr10:3401033 | *SuIAA11* | chr02:18809276 | 0.38 | 3.09 | 0.12 | segmental |
| *SuIAA13* | chr08:9718353 | *SuIAA11* | chr02:18809276 | 0.38 | 3.83 | 0.1 | segmental |
| *SuIAA33.1* | chr18:9204456 | *SuIAA33.2* | chr06:12154904 | 0.15 | 0.27 | 0.55 | segmental |
| *SuIAA3.4* | chr05:16238089 | *SuIAA3.1* | chr05:3155171 | 0.25 | 3.63 | 0.07 | segmental |
| *SuIAA3.6* | chr10:5680397 | *SuIAA3.1* | chr05:3155171 | 0.26 | 1.38 | 0.19 | segmental |
| *SuIAA34* | chr10:8413834 | *SuIAA12.1* | chr10:3401033 | 0.59 | 3.36 | 0.17 | transposed |
| *SuIAA27.3* | chr16:14555673 | *SuIAA9* | chr02:7289305 | 0.36 | 4.02 | 0.09 | transposed |
| *VviIAA15* | chr4:163293 | *VviIAA19* | chr9:3076002 | 0.29 | 1.38 | 0.21 | segmental |
| *VviIAA20* | chr5:2845731 | *VviIAA7* | chr7:4888143 | 0.28 | 2.57 | 0.11 | segmental |
| *VviIAA22* | chr5:6536421 | *VviIAA6* | chr7:165826 | 0.17 | 1.08 | 0.15 | segmental |
| *VviIAA21* | chr5:6548474 | *VviIAA8* | chr7:180388 | 0.12 | 1.71 | 0.07 | segmental |
| *VviIAA23* | chr5:9458989 | *VviIAA9* | chr7:7467843 | 0.25 | 2.74 | 0.09 | segmental |
| *VviIAA17* | chr9:3750452 | *VviIAA13* | chr11:2881794 | 0.17 | 1.33 | 0.13 | segmental |
| *VviIAA14* | chr4:5036148 | *VviIAA11* | chr11:5096181 | 0.41 | 2.14 | 0.19 | segmental |
| *VviIAA22* | chr5:6536421 | *VviIAA1* | chr14:7536603 | 0.17 | 1.08 | 0.16 | segmental |
| *VviIAA6* | chr7:165826 | *VviIAA1* | chr14:7536603 | 0.15 | 0.77 | 0.2 | segmental |
| *VviIAA21* | chr5:6548474 | *VviIAA2* | chr14:7563482 | 0.13 | 2.86 | 0.04 | segmental |
| *VviIAA8* | chr7:180388 | *VviIAA2* | chr14:7563482 | 0.14 | 2.03 | 0.07 | segmental |
| *VviIAA10* | chr11:3794574 | *VviIAA2* | chr14:7563482 | 0.36 | 3.73 | 0.1 | transposed |
| *VviIAA8* | chr7:180388 | *VviIAA4* | chr18:6527840 | 0.39 | 3.8 | 0.1 | transposed |
| *VviIAA17* | chr9:3750452 | *VviIAA5* | chr18:11789356 | 0.94 | 2.48 | 0.38 | transposed |
| *VviIAA6* | chr7:165826 | *VviIAA8* | chr7:180388 | 0.39 | 3.61 | 0.11 | tandem |
| *OsaIAA4* | Chr1:10312346 | *OsaIAA13* | Chr2:30064481 | 0.28 | 2.04 | 0.14 | transposed |
| *OsaIAA6* | Chr1:7249761 | *OsaIAA15* | Chr2:35073371 | 0.63 | 3.85 | 0.16 | transposed |
| *OsaIAA6* | Chr1:7249761 | *OsaIAA21* | Chr6:13345333 | 0.23 | 3.19 | 0.07 | transposed |
| *OsaIAA14* | Chr2:7245015 | *OsaIAA20* | Chr6:14579528 | 0.59 | 1.76 | 0.33 | transposed |
| *OsaIAA7* | Chr1:4073676 | *OsaIAA19* | Chr6:23501973 | 0.21 | 1.26 | 0.17 | transposed |
| *OsaIAA14* | Chr2:7245015 | *OsaIAA1* | Chr8:471199 | 0.62 | 2.64 | 0.23 | transposed |
| *OsaIAA17* | Chr9:20641465 | *OsaIAA23* | Chr12:25310812 | 0.35 | 3.14 | 0.11 | transposed |
| *OsaIAA30* | Chr11:6335893 | *OsaIAA29* | Chr11:6348492 | 0.74 | 1.43 | 0.51 | tandem |
| *OsaIAA29* | Chr11:6348492 | *OsaIAA31* | Chr11:6356615 | 0.13 | 0.26 | 0.48 | tandem |
| *OsaIAA7* | Chr1:4073676 | *OsaIAA27* | Chr5:4681460 | 0.12 | 0.9 | 0.13 | segmental |
| *OsaIAA8* | Chr1:4816845 | *OsaIAA25* | Chr5:5329581 | 0.22 | 0.68 | 0.33 | segmental |
| *OsaIAA6* | Chr1:7249761 | *OsaIAA28* | Chr5:7950872 | 0.09 | 0.74 | 0.12 | segmental |
| *OsaIAA3* | Chr1:27777506 | *OsaIAA24* | Chr5:27845887 | 0.17 | 0.87 | 0.19 | segmental |
| *OsaIAA5* | Chr1:30975765 | *OsaIAA26* | Chr5:26052916 | 0.28 | 0.86 | 0.32 | segmental |
| *OsaIAA16* | Chr2:34348582 | *OsaIAA18* | Chr6:3342423 | 0.43 | 3.38 | 0.13 | segmental |
| *OsaIAA12* | Chr3:30480662 | *OsaIAA9* | Chr3:33246011 | 0.39 | 3.49 | 0.11 | segmental |
| *OsaIAA9* | Chr3:33246011 | *OsaIAA2* | Chr7:4355211 | 0.27 | 2.09 | 0.13 | segmental |
| *OsaIAA11* | Chr3:24198649 | *OsaIAA23* | Chr12:25310812 | 0.15 | 2.59 | 0.06 | segmental |
| *OsaIAA10* | Chr3:24212554 | *OsaIAA22* | Chr12:25325549 | 0.19 | 1.72 | 0.11 | segmental |
| *AtrIAA2* | AmTr_v1.0_scaffold00002:8774435 | *AtrIAA3* | AmTr_v1.0_scaffold00002:8794997 | 0.34 | 3.64 | 0.09 | proximal |
| *SmoIAA2* | scaffold_18:1264140 | *SmoIAA4* | scaffold_6:60600 | 0.28 | 3.39 | 0.08 | transposed |
| *SmoIAA7* | scaffold_53:774450 | *SmoIAA1* | scaffold_7:1431872 | 0.03 | 0.16 | 0.18 | segmental |
| *SmoIAA1* | scaffold_7:1431872 | *SmoIAA4* | scaffold_6:60600 | 0.21 | 2.26 | 0.09 | segmental |
| *PpaIAA1* | Chr24:4415762 | *PpaIAA2* | Chr08:9600165 | 0.18 | 1.46 | 0.12 | segmental |

**Table S5.** The statistics of Ka, Ks, and Ka/Ks values for segmental, tandem, proximal and transposed duplicated ARF genes in the genomes of *A. thaliana*, *P. trichocarpa*, *S. suchowensis*, *V. vinifera*, *O. sativia*, *A. trichoposa*, *S. moellendorffii*, and *P. patens*.

| Duplicated1 | Location 1 | Duplicated2 | Location 2 | Ka | Ks | Ka/Ks | Mode |
| --- | --- | --- | --- | --- | --- | --- | --- |
| *AthARF11* | Chr2:19105112 | *AthARF18* | Chr3:22887889 | 0.18 | 0.97 | 0.19 | segmental |
| *AthARF11* | Chr2:19105112 | *AthARF9* | Chr4:12451143 | 0.34 | 4.49 | 0.08 | segmental |
| *AthARF18* | Chr3:22887889 | *AthARF9* | Chr4:12451143 | 0.34 | 4.46 | 0.08 | segmental |
| *AthARF22* | Chr1:12556005 | *AthARF21* | Chr1:12577722 | 0.05 | 0.08 | 0.65 | proximal |
| *AthARF15* | Chr1:13082819 | *AthARF14* | Chr1:13108634 | 0.06 | 0.1 | 0.65 | proximal |
| *AthARF1* | Chr1:21979339 | *AthARF2* | Chr5:24910209 | 0.36 | 4.59 | 0.08 | transposed |
| *AthARF17* | Chr1:29272205 | *AthARF16* | Chr4:14703065 | 0.63 | 4.49 | 0.14 | transposed |
| *AthARF7* | Chr5:7016470 | *AthARF8* | Chr5:14629453 | 0.42 | 4.68 | 0.09 | transposed |
| *PtrARF2.3* | Chr01:5248897 | *PtrARF2.4* | Chr03:17441249 | 0.08 | 0.2 | 0.4 | segmental |
| *PtrARF2.3* | Chr01:5248897 | *PtrARF2.1* | Chr12:12992423 | 0.32 | 1.81 | 0.18 | segmental |
| *PtrARF2.3* | Chr01:5248897 | *PtrARF2.2* | Chr15:12321925 | 0.31 | 1.96 | 0.16 | segmental |
| *PtrARF9.2* | Chr01:6994446 | *PtrARF9.1* | Chr03:15883566 | 0.05 | 0.24 | 0.21 | segmental |
| *PtrARF9.2* | Chr01:6994446 | *PtrARF9.4* | Chr14:7830151 | 0.23 | 1.64 | 0.14 | segmental |
| *PtrARF6.1* | Chr01:36824007 | *PtrARF6.2* | Chr11:11137606 | 0.06 | 0.17 | 0.35 | segmental |
| *PtrARF5.1* | Chr02:1554518 | *PtrARF5.2* | Chr05:24385396 | 0.05 | 0.21 | 0.24 | segmental |
| *PtrARF6.4* | Chr02:3666161 | *PtrARF6.5* | Chr05:22191499 | 0.04 | 0.18 | 0.21 | segmental |
| *PtrARF17.1* | Chr02:6401227 | *PtrARF17.2* | Chr05:18573573 | 0.06 | 0.25 | 0.24 | segmental |
| *PtrARF2.6* | Chr02:17278044 | *PtrARF2.6* | Chr14:10298617 | 0.09 | 0.2 | 0.45 | segmental |
| *PtrARF1.1* | Chr03:191625 | *PtrARF1.2* | Chr04:23374101 | 0.05 | 0.26 | 0.18 | segmental |
| *PtrARF9.1* | Chr03:15883566 | *PtrARF9.4* | Chr14:7830151 | 0.23 | 1.63 | 0.14 | segmental |
| *PtrARF2.4* | Chr03:17441249 | *PtrARF2.1* | Chr12:12992423 | 0.33 | 1.55 | 0.21 | segmental |
| *PtrARF2.4* | Chr03:17441249 | *PtrARF2.2* | Chr15:12321925 | 0.31 | 2.09 | 0.15 | segmental |
| *PtrARF8.1* | Chr04:6461508 | *PtrARF8.2* | Chr17:14855388 | 0.03 | 0.19 | 0.16 | segmental |
| *PtrARF10.1* | Chr04:21930073 | *PtrARF10.2* | Chr09:2531890 | 0.09 | 0.25 | 0.35 | segmental |
| *PtrARF7.4* | Chr06:5883110 | *PtrARF7.3* | Chr18:16534350 | 0.04 | 0.17 | 0.26 | segmental |
| *PtrARF16.4* | Chr06:10401867 | *PtrARF16.2* | Chr08:2208660 | 0.22 | 1.53 | 0.14 | segmental |
| *PtrARF16.4* | Chr06:10401867 | *PtrARF16.1* | Chr10:20732887 | 0.21 | 1.89 | 0.11 | segmental |
| *PtrARF16.4* | Chr06:10401867 | *PtrARF16.3* | Chr16:7359296 | 0.08 | 0.22 | 0.36 | segmental |
| *PtrARF16.2* | Chr08:2208660 | *PtrARF16.1* | Chr10:20732887 | 0.05 | 0.21 | 0.22 | segmental |
| *PtrARF16.2* | Chr08:2208660 | *PtrARF16.3* | Chr16:7359296 | 0.24 | 2.24 | 0.11 | segmental |
| *PtrARF16.1* | Chr10:20732887 | *PtrARF16.3* | Chr16:7359296 | 0.23 | 1.64 | 0.14 | segmental |
| *PtrARF2.1* | Chr12:12992423 | *PtrARF2.2* | Chr15:12321925 | 0.06 | 0.25 | 0.25 | segmental |
| *SuARF2.3* | chr16:3798407 | *SuARF2.5* | chr16:3832851 | 0.01 | 0.02 | 0.76 | proximal |
| *SuARF9.1* | chr03:9946266 | *SuARF9.2* | chr16:5349356 | 0.08 | 0.25 | 0.33 | segmental |
| *SuARF9.1* | chr03:9946266 | *SuARF9.4* | chr14:6114500 | 0.24 | 1.71 | 0.14 | segmental |
| *SuARF6.4* | chr05:15406241 | *SuARF6.3* | chr02:3458978 | 0.06 | 0.27 | 0.22 | segmental |
| *SuARF1.1* | chr03:56472 | *SuARF1.2* | chr16:16174273 | 0.07 | 0.29 | 0.24 | segmental |
| *SuARF9.1* | chr03:9946266 | *SuARF9.3* | chr02:13643406 | 0.22 | 1.51 | 0.15 | segmental |
| *SuARF9.3* | chr02:13643406 | *SuARF9.2* | chr16:5349356 | 0.24 | 1.57 | 0.16 | segmental |
| *SuARF9.3* | chr02:13643406 | *SuARF9.4* | chr14:6114500 | 0.07 | 0.25 | 0.29 | segmental |
| *SuARF5.1* | chr02:1491797 | *SuARF5.2* | chr05:17637399 | 0.06 | 0.34 | 0.16 | segmental |
| *SuARF7.3* | chr06:4794509 | *SuARF7.2* | chr18:13189520 | 0.06 | 0.22 | 0.28 | segmental |
| *SuARF2.3* | chr16:3798407 | *SuARF2.1* | chr12:8444211 | 0.34 | 2.18 | 0.15 | segmental |
| *SuARF2.1* | chr12:8444211 | *SuARF2.2* | chr15:10663901 | 0.09 | 0.37 | 0.25 | segmental |
| *SuARF2.3* | chr16:3798407 | *SuARF2.4* | chr03:11214039 | 0.1 | 0.28 | 0.37 | segmental |
| *SuARF2.1* | chr12:8444211 | *SuARF2.4* | chr03:11214039 | 0.31 | 2.29 | 0.14 | segmental |
| *SuARF2.4* | chr03:11214039 | *SuARF2.2* | chr15:10663901 | 0.33 | 2.45 | 0.13 | segmental |
| *SuARF3.2* | chr11:3800600 | *SuARF3.1* | chr04:2696603 | 0.1 | 0.3 | 0.35 | segmental |
| *SuARF10.2* | chr09:812342 | *SuARF16.4* | chr06:8731536 | 0.28 | 1.63 | 0.17 | segmental |
| *SuARF16.1* | chr10:15132192 | *SuARF16.4* | chr06:8731536 | 0.22 | 1.54 | 0.14 | segmental |
| *SuARF17.2* | chr05:12147208 | *SuARF17.1* | chr02:5914597 | 0.1 | 0.33 | 0.3 | segmental |
| *SuARF6.2* | chr11:9689639 | *SuARF6.1* | chr01:25251650 | 0.09 | 0.26 | 0.35 | segmental |
| *SuARF16.1* | chr10:15132192 | *SuARF16.3* | chr01:9603011 | 0.25 | 1.49 | 0.17 | segmental |
| *SuARF16.4* | chr06:8731536 | *SuARF16.3* | chr01:9603011 | 0.1 | 0.34 | 0.28 | segmental |
| *SuARF16.3* | chr01:9603011 | *SuARF10.1* | chr04:16073742 | 0.3 | 2.07 | 0.15 | segmental |
| *SuARF16.3* | chr01:9603011 | *SuARF16.2* | chr08:1948787 | 0.26 | 3.03 | 0.09 | segmental |
| *SuARF2.3* | chr16:3798407 | *SuARF2.2* | chr15:10663901 | 0.35 | 1.86 | 0.19 | segmental |
| *SuARF10.2* | chr09:812342 | *SuARF10.1* | chr04:16073742 | 0.11 | 0.34 | 0.34 | segmental |
| *SuARF16.4* | chr06:8731536 | *SuARF10.1* | chr04:16073742 | 0.28 | 2.69 | 0.11 | segmental |
| *SuARF9.2* | chr16:5349356 | *SuARF9.4* | chr14:6114500 | 0.24 | 1.72 | 0.14 | segmental |
| *SuARF10.2* | chr09:812342 | *SuARF16.2* | chr08:1948787 | 0.25 | 1.83 | 0.14 | segmental |
| *SuARF16.1* | chr10:15132192 | *SuARF16.2* | chr08:1948787 | 0.05 | 0.34 | 0.15 | segmental |
| *SuARF8.2* | chr17:13163854 | *SuARF8.1* | chr04:4334989 | 0.05 | 0.25 | 0.22 | segmental |
| *VViARF16* | chr6:3443778 | *VViARF1* | chr8:12924872 | 0.19 | 1.65 | 0.12 | segmental |
| *VViARF16* | chr6:3443778 | *VViARF12* | chr13:5744554 | 0.17 | 1.05 | 0.16 | segmental |
| *VViARF1* | chr8:12924872 | *VViARF12* | chr13:5744554 | 0.2 | 1.27 | 0.16 | segmental |
| *VViARF11* | chrUn:37807086 | *VViARF8* | chr12:1746885 | 0.14 | 1.47 | 0.1 | transposed |
| *VViARF20* | chr4:10403355 | *VViARF8* | chr12:1746885 | 0.11 | 3.7 | 0.03 | transposed |
| *VViARF21* | chr4:10435489 | *VViARF8* | chr12:1746885 | 0.28 | 4.54 | 0.06 | transposed |
| *VViARF14* | chr10:6956699 | *VViARF18* | chr11:629738 | 0.42 | 4.77 | 0.09 | transposed |
| *VViARF10* | chr2:1653905 | *VViARF5* | chr18:28750309 | 0.32 | 4.52 | 0.07 | transposed |
| *OsaARF4* | Chr4:33888004 | *OsaARF20* | Chr6:28158507 | 0.58 | 4.79 | 0.12 | transposed |
| *OsaARF16* | Chr2:3486813 | *OsaARF5* | Chr4:34278412 | 0.3 | 4.79 | 0.06 | transposed |
| *OsaARF6* | Chr4:26013418 | *OsaARF17* | Chr6:28586445 | 0.3 | 4.45 | 0.07 | transposed |
| *OsaARF18* | Chr6:4926492 | *OsaARF2* | Chr8:25870712 | 0.42 | 4.89 | 0.09 | transposed |
| *OsaARF16* | Chr2:3486813 | *OsaARF22* | Chr12:26002359 | 0.27 | 2.18 | 0.13 | transposed |
| *OsaARF7* | Chr7:4403712 | *OsaARF9* | Chr7:4406706 | 0.12 | 0.26 | 0.47 | tandem |
| *OsaARF13* | Chr1:27501563 | *OsaARF11* | Chr1:31617167 | 0.46 | 1.12 | 0.42 | segmental |
| *OsaARF13* | Chr1:27501563 | *OsaARF23* | Chr5:28034380 | 0.19 | 0.59 | 0.32 | segmental |
| *OsaARF11* | Chr1:31617167 | *OsaARF24* | Chr5:25555850 | 0.29 | 0.68 | 0.43 | segmental |
| *OsaARF16* | Chr2:3486813 | *OsaARF20* | Chr6:28158507 | 0.09 | 0.88 | 0.1 | segmental |
| *OsaARF14* | Chr2:21098150 | *OsaARF3* | Chr4:22006216 | 0.13 | 0.87 | 0.15 | segmental |
| *OsaARF15* | Chr2:25133442 | *OsaARF6* | Chr4:26013418 | 0.18 | 1.53 | 0.12 | segmental |
| *OsaARF15* | Chr2:25133442 | *OsaARF1* | Chr10:18051860 | 0.25 | 4.46 | 0.06 | segmental |
| *OsaARF6* | Chr4:26013418 | *OsaARF1* | Chr10:18051860 | 0.34 | 4.49 | 0.08 | segmental |
| *OsaARF8* | Chr7:4400490 | *OsaARF9* | Chr7:4406706 | 0.65 | 2.08 | 0.31 | proximal |
| *AtrARF11* | AmTr_v1.0_scaffold00016:2703406 | *AtrARF12* | AmTr_v1.0_scaffold00017:3105775 | 0.83 | 4.54 | 0.18 | transposed |
| *AtrARF11* | AmTr_v1.0_scaffold00016:2703406 | *AtrARF7* | AmTr_v1.0_scaffold00021:3709558 | 0.58 | 4.72 | 0.12 | transposed |
| *AtrARF6* | AmTr_v1.0_scaffold00021:4517273 | *AtrARF14* | AmTr_v1.0_scaffold00057:2033729 | 0.41 | 4.59 | 0.09 | transposed |
| *AtrARF1* | AmTr_v1.0_scaffold00029:2410874 | *AtrARF14* | AmTr_v1.0_scaffold00057:2033729 | 0.6 | 4.68 | 0.13 | transposed |
| *AtrARF14* | AmTr_v1.0_scaffold00057:2033729 | *AtrARF2* | AmTr_v1.0_scaffold00092:780143 | 0.54 | 4.67 | 0.12 | transposed |
| *AtrARF11* | AmTr_v1.0_scaffold00016:2703406 | *AtrARF3* | AmTr_v1.0_scaffold00148:409592 | 0.76 | 4.6 | 0.17 | transposed |
| *AtrARF14* | AmTr_v1.0_scaffold00057:2033729 | *AtrARF8* | AmTr_v1.0_scaffold00155:699229 | 0.68 | 4.14 | 0.17 | transposed |
| *AtrARF11* | AmTr_v1.0_scaffold00016:2703406 | *AtrARF14* | AmTr_v1.0_scaffold00057:2033729 | 0.3 | 4.34 | 0.07 | segmental |
| *SmoARF1* | scaffold_55:224397 | *SmoARF4* | scaffold_65:583786 | 0.22 | 4.28 | 0.05 | transposed |
| *SmoARF4* | scaffold_65:583786 | *SmoARF6* | scaffold_1:4121931 | 0.56 | 4.68 | 0.12 | transposed |
| *SmoARF4* | scaffold_65:583786 | *SmoARF3* | scaffold_4:1853623 | 0.35 | 4.8 | 0.07 | transposed |
| *PpaARF11* | Chr01:10658024 | *PpaARF6* | Chr02:17539856 | 0.03 | 0.4 | 0.07 | segmental |
| *PpaARF5* | Chr13:2898132 | *PpaARF6* | Chr02:17539856 | 0.23 | 2.07 | 0.11 | transposed |
| *PpaARF6* | Chr02:17539856 | *PpaARF13* | Chr06:17685212 | 0.75 | 4.57 | 0.16 | transposed |
| *PpaARF3* | Chr04:9083311 | *PpaARF4* | Chr04:9092542 | 0 | 0.01 | 0.05 | proximal |
| *PpaARF11* | Chr01:10658024 | *PpaARF10* | Chr01:10698060 | 0 | 0 | 1.03 | tandem |

**Table S6.** The time-sequential transcriptome profiles of SuIAAs and SuARFs in the cambium tissues of two contrasting clones “S328” and “S3412”

|  | S3412 | | | | | | S328 | | | | | |
| --- | --- | --- | --- | --- | --- | --- | --- | --- | --- | --- | --- | --- |
| Genes | 45 days | 75 days | 135 days | 195 days | 240 days | 270 days | 45 days | 75 days | 135 days | 195 days | 240 days | 270 days |
| *SuIAA11* | 42.4 | 38.4 | 30.4 | 32.1 | 22.7 | 62.7 | 49.3 | 34.9 | 25.3 | 22.6 | 22.2 | 76.6 |
| *SuIAA12.1* | 26.7 | 17.7 | 28.8 | 23.8 | 22.7 | 19.6 | 21.6 | 14.8 | 25.7 | 18.3 | 17.4 | 35.6 |
| *SuIAA12.2* | 6.4 | 21.8 | 26.4 | 20.1 | 18.9 | 18.1 | 6.8 | 15.2 | 11.2 | 15.6 | 14.2 | 18.7 |
| *SuIAA13* | 1.9 | 7.4 | 8.5 | 6.1 | 6.4 | 5.7 | 2.2 | 5.6 | 3.8 | 6.1 | 4.8 | 6.4 |
| *SuIAA15* | 238.6 | 57.9 | 90.8 | 75.2 | 95.2 | 53 | 226.5 | 59.1 | 82 | 49 | 68.3 | 67.9 |
| *SuIAA16.1* | 228.8 | 202.4 | 148 | 139.8 | 132.8 | 81.9 | 260.7 | 187.3 | 97 | 125.3 | 122.1 | 89.6 |
| *SuIAA16.2* | 69.3 | 132.9 | 106 | 123.9 | 138.1 | 64.5 | 71.6 | 123.2 | 84 | 94.2 | 123.3 | 80 |
| *SuIAA16.3* | 0 | 0 | 0 | 0 | 0 | 0.1 | 27.4 | 0.2 | 0 | 0 | 0 | 0 |
| *SuIAA16.4* | 20 | 2 | 2.4 | 1.2 | 1.6 | 1.2 | 41.6 | 3.1 | 1.2 | 0.7 | 1.2 | 1.1 |
| *SuIAA16.5* | 136.1 | 216.4 | 172.5 | 195.1 | 225.8 | 109.9 | 144.8 | 192.9 | 138 | 153.4 | 196.3 | 128.2 |
| *SuIAA19.1* | 49.2 | 0 | 1 | 0.6 | 1 | 0.2 | 25.1 | 0.6 | 2.5 | 1.5 | 0.9 | 0.9 |
| *SuIAA19.2* | 12.2 | 0.4 | 0.1 | 0 | 0.3 | 0 | 18.9 | 0.3 | 0.2 | 0 | 0.1 | 0 |
| *SuIAA20.1* | 3.8 | 23.5 | 36 | 26.4 | 43.6 | 20.6 | 2.7 | 5.8 | 23.3 | 28.3 | 50.3 | 44 |
| *SuIAA20.2* | 6.1 | 27.6 | 24.3 | 50.3 | 32.1 | 48.7 | 6.6 | 17.4 | 17.6 | 17.9 | 46.5 | 42.2 |
| *SuIAA26* | 33.2 | 19.8 | 17.9 | 20.6 | 20.8 | 4.8 | 19.6 | 32.5 | 29.3 | 38.3 | 28.8 | 9.8 |
| *SuIAA27.1* | 17.3 | 12.5 | 9.8 | 23.3 | 22.6 | 8.4 | 57.5 | 65.5 | 62.9 | 70.8 | 75.5 | 41.6 |
| *SuIAA27.2* | 21 | 16.9 | 12 | 15.7 | 18.3 | 5.5 | 28.1 | 15.4 | 13.2 | 20.5 | 19.8 | 10.7 |
| *SuIAA27.3* | 127.7 | 76 | 60.6 | 120.7 | 130.2 | 57.5 | 27.8 | 33.4 | 29.2 | 34.2 | 36.1 | 19.8 |
| *SuIAA28* | 24.6 | 152.3 | 46.7 | 49.1 | 41.5 | 37.9 | 22.8 | 94 | 30.1 | 33.2 | 45.3 | 57.3 |
| *SuIAA29.1* | 6.5 | 13.2 | 13.5 | 11 | 16.7 | 5.3 | 4 | 4 | 8.2 | 10.1 | 12.1 | 16.5 |
| *SuIAA29.2* | 0.9 | 1.7 | 5.9 | 4.3 | 3.4 | 6.2 | 2.6 | 5.6 | 5.6 | 3.9 | 2.8 | 4 |
| *SuIAA29.3* | 0.3 | 0 | 0.8 | 0.8 | 0.9 | 2.1 | 0.5 | 0.5 | 0.8 | 0.7 | 1.5 | 2 |
| *SuIAA29.4* | 1.8 | 7.7 | 15.4 | 9.1 | 12.2 | 3.4 | 2.4 | 2.3 | 3.8 | 5.8 | 6.9 | 6.1 |
| *SuIAA29.5* | 2.7 | 11.6 | 22.8 | 13.2 | 17.9 | 5.2 | 3.5 | 3.4 | 5.6 | 8.6 | 10.3 | 9 |
| *SuIAA3.1* | 253.5 | 430.9 | 337.1 | 382 | 527.8 | 224.7 | 284.2 | 359.2 | 226.8 | 347.6 | 457.6 | 322.4 |
| *SuIAA3.2* | 20.3 | 26.4 | 30.3 | 27.9 | 47.5 | 18.5 | 39.7 | 27.6 | 35.4 | 33.7 | 49.8 | 29.3 |
| *SuIAA3.3* | 37.8 | 0.2 | 0.5 | 0.2 | 0.3 | 0.2 | 42.4 | 0.5 | 0.5 | 0.1 | 0.2 | 0.2 |
| *SuIAA3.4* | 10.6 | 0 | 0.1 | 0 | 0.3 | 0.1 | 15.8 | 0 | 0.1 | 0 | 0.2 | 0.2 |
| *SuIAA3.5* | 58 | 36.7 | 89.5 | 36.1 | 47.7 | 85 | 64.7 | 39.4 | 51.8 | 30.6 | 46.1 | 104.5 |
| *SuIAA3.6* | 11.3 | 4.3 | 28.7 | 27.7 | 23.1 | 39.4 | 15.7 | 5.4 | 22.4 | 10.2 | 14.2 | 31.3 |
| *SuIAA3.7* | 88.5 | 110.2 | 126.3 | 118.1 | 190.2 | 81.6 | 73.3 | 52.2 | 64.2 | 61.8 | 83.5 | 52 |
| *SuIAA33.1* | 0 | 0 | 0 | 0.1 | 0 | 0 | 0 | 0.1 | 0 | 0 | 0 | 0 |
| *SuIAA33.2* | 0.1 | 0 | 0.1 | 0.1 | 0.1 | 0 | 0 | 0.1 | 0.1 | 0.1 | 0.2 | 0 |
| *SuIAA33.3* | 0 | 0 | 0 | 0 | 0 | 0 | 0 | 0.4 | 0 | 0 | 0 | 0 |
| *SuIAA34* | 0.1 | 0.2 | 0.2 | 0.6 | 0.4 | 0 | 0 | 0 | 0 | 0 | 0 | 0 |
| *SuIAA7.1* | 1 | 0 | 0.3 | 0.4 | 0.3 | 0.3 | 1.8 | 0.2 | 0.3 | 0.5 | 0.4 | 0.8 |
| *SuIAA7.2* | 23.2 | 0.2 | 0.3 | 0.1 | 0.1 | 0.1 | 36.8 | 0.9 | 0.4 | 0.1 | 0.1 | 0 |
| *SuIAA9* | 282.5 | 155.2 | 160.1 | 163.9 | 156.9 | 96.4 | 305.6 | 201.1 | 173.1 | 218.2 | 164.9 | 112.9 |
| *SuARF1.1* | 25.1 | 34.4 | 38.3 | 27.6 | 24.3 | 46.6 | 28.9 | 30.3 | 26.3 | 28.8 | 25.2 | 36.3 |
| *SuARF1.2* | 9.8 | 14.6 | 24.2 | 17.7 | 16.2 | 30.5 | 11.9 | 12.6 | 15.7 | 17.8 | 17.3 | 27.4 |
| *SuARF10.1* | 9 | 2.7 | 3.4 | 3.2 | 3.9 | 2.4 | 8 | 3.4 | 4.5 | 4.9 | 4.3 | 4 |
| *SuARF10.2* | 10.1 | 3 | 2.8 | 2.8 | 2.8 | 3 | 11.3 | 4.1 | 3.7 | 3.7 | 3.2 | 4.3 |
| *SuARF16.1* | 11.5 | 3 | 4.6 | 6.6 | 3.1 | 8.3 | 14.2 | 4.3 | 3.4 | 3.8 | 2.6 | 8.6 |
| *SuARF16.2* | 7.2 | 2.3 | 3.6 | 3.5 | 2.8 | 5.9 | 8.5 | 2.7 | 2.9 | 2.2 | 2.8 | 5.6 |
| *SuARF16.3* | 1.3 | 2.5 | 2.8 | 3.3 | 2.7 | 1.9 | 1.6 | 3.8 | 3.6 | 3.8 | 3.1 | 2.9 |
| *SuARF16.4* | 0.9 | 1.1 | 0.7 | 1.1 | 0.8 | 2.8 | 1.7 | 1.9 | 0.8 | 1.2 | 0.9 | 2.7 |
| *SuARF17.1* | 4.4 | 2.6 | 3.6 | 3.2 | 2.5 | 4.6 | 4.6 | 3.4 | 3.5 | 3.8 | 2.2 | 3.7 |
| *SuARF17.2* | 3.5 | 2.5 | 2.5 | 1.7 | 1.7 | 2.6 | 2.2 | 2.4 | 1.6 | 1.5 | 0.9 | 1.8 |
| *SuARF2.1* | 18.5 | 34.7 | 46.6 | 44.9 | 48.8 | 153.7 | 30.1 | 30.4 | 33 | 34.9 | 54.9 | 119.4 |
| *SuARF2.2* | 49.8 | 116.4 | 127.5 | 90.9 | 135.8 | 120.1 | 62.2 | 81.5 | 100.9 | 109.8 | 137.5 | 105.9 |
| *SuARF2.3* | 2.8 | 4.7 | 3.3 | 6 | 10.1 | 12.9 | 2.5 | 2.6 | 4 | 5.3 | 10.7 | 13.8 |
| *SuARF2.4* | 1.8 | 3.2 | 1.1 | 1.4 | 0.9 | 0.3 | 1.5 | 2.7 | 2.5 | 2.9 | 1 | 0.3 |
| *SuARF2.5* | 1.8 | 3.4 | 2.2 | 3.1 | 6.7 | 8.9 | 1.6 | 1.7 | 2.6 | 3.6 | 7.1 | 9.3 |
| *SuARF2.6* | 3.2 | 3.5 | 3.7 | 2.4 | 3.2 | 3.6 | 3.3 | 3.6 | 3.2 | 2.8 | 2.7 | 3.3 |
| *SuARF3.1* | 18.4 | 12 | 12.4 | 17.5 | 15.9 | 33.2 | 15.6 | 17.9 | 19 | 16.3 | 15 | 25.1 |
| *SuARF3.2* | 14.2 | 23.7 | 17.1 | 14.3 | 16.7 | 20.5 | 12.8 | 31 | 22.8 | 17.5 | 16.2 | 22 |
| *SuARF4* | 25.7 | 23.5 | 25.9 | 21.7 | 29.6 | 39.2 | 32.1 | 33.4 | 32.7 | 24.2 | 25.7 | 32 |
| *SuARF5.1* | 2.6 | 8.6 | 5.1 | 5.8 | 5.5 | 2.3 | 2.4 | 11.5 | 10.6 | 16.9 | 11 | 4.4 |
| *SuARF5.2* | 8.2 | 4.4 | 5.9 | 5.1 | 9.6 | 2.5 | 6.3 | 13.2 | 13.1 | 22.2 | 16.9 | 6.4 |
| *SuARF6.1* | 12.9 | 15.4 | 10.8 | 8.3 | 15.5 | 17.7 | 21.2 | 21.3 | 19.8 | 24.1 | 28.5 | 27.5 |
| *SuARF6.2* | 17.4 | 8.7 | 7.7 | 6.4 | 10.6 | 16.7 | 16.6 | 10.4 | 9.4 | 9.3 | 10.3 | 15.4 |
| *SuARF6.3* | 28.4 | 44.7 | 43.1 | 25.4 | 31.1 | 14 | 29.6 | 44.4 | 36.5 | 39.6 | 31.4 | 13.4 |
| *SuARF6.4* | 29.9 | 77.3 | 70.6 | 52.7 | 46.9 | 48.2 | 26.5 | 79 | 63.7 | 71 | 40.2 | 45.6 |
| *SuARF7.1* | 28.4 | 23.3 | 25.2 | 13.7 | 19.9 | 16.1 | 49.2 | 23.2 | 19.6 | 18.2 | 17.7 | 14 |
| *SuARF7.2* | 12.2 | 22.8 | 46.3 | 22.6 | 23.8 | 14.6 | 11.9 | 26.3 | 25.1 | 32 | 18.6 | 19.2 |
| *SuARF7.3* | 20.5 | 36.8 | 33.8 | 23.8 | 24.4 | 31.8 | 23 | 36.9 | 31.6 | 39.5 | 27.4 | 36.8 |
| *SuARF8.1* | 35.3 | 41.4 | 27 | 22.2 | 44.6 | 78.4 | 44.3 | 38.1 | 27.3 | 35.1 | 37.9 | 73.1 |
| *SuARF8.2* | 14.1 | 12.8 | 11.1 | 8.3 | 9.3 | 7.8 | 12.5 | 13.2 | 12.3 | 13 | 9.2 | 9.9 |
| *SuARF9.1* | 10.2 | 14.9 | 16.1 | 15.7 | 17 | 19.5 | 11.1 | 21.3 | 11.2 | 12.4 | 11.6 | 23.1 |
| *SuARF9.2* | 15.6 | 24.1 | 31 | 28.8 | 27.6 | 40.3 | 18.3 | 26 | 22.5 | 26.1 | 29.3 | 39 |
| *SuARF9.3* | 4 | 22.1 | 24.8 | 22.6 | 20.6 | 87 | 8.1 | 23.1 | 21.6 | 27.3 | 33.5 | 89.9 |
| *SuARF9.4* | 10.7 | 11.8 | 18.9 | 24.3 | 26.3 | 58.3 | 7.9 | 8.2 | 20.6 | 27 | 33.2 | 62.4 |
